# An anti-tuberculosis compound screen using a zebrafish infection model identifies an aspartyl-tRNA synthetase inhibitor

**DOI:** 10.1242/dmm.049145

**Published:** 2021-12-23

**Authors:** Eva Habjan, Vien Q. T. Ho, James Gallant, Gunny van Stempvoort, Kin Ki Jim, Coen Kuijl, Daan P. Geerke, Wilbert Bitter, Alexander Speer

**Affiliations:** 1 Department of Medical Microbiology and Infection Control, Amsterdam UMC, Location Vrije Universiteit Medical Center, De Boelelaan 1108, 1081 HZ Amsterdam, The Netherlands; 2 Section Molecular Microbiology, Amsterdam Institute of Molecular and Life Sciences, Vrije Universiteit Amsterdam, De Boelelaan 1108, 1081 HZ Amsterdam, The Netherlands; 3 Department of Molecular Toxicology, Amsterdam Institute of Molecular and Life Sciences, Vrije Universiteit Amsterdam, De Boelelaan 1108, 1081 HZ Amsterdam, The Netherlands

**Keywords:** *Mycobacterium tuberculosis*, Tuberculosis, Zebrafish, Infection model, Drug screening, Aminoacyl-tRNA synthetase

## Abstract

Finding new anti-tuberculosis compounds with convincing *in vivo* activity is an ongoing global challenge to fight the emergence of multidrug-resistant *Mycobacterium tuberculosis* isolates. In this study, we exploited the medium-throughput capabilities of the zebrafish embryo infection model with *Mycobacterium marinum* as a surrogate for *M. tuberculosis*. Using a representative set of clinically established drugs, we demonstrate that this model could be predictive and selective for antibiotics that can be administered orally. We further used the zebrafish infection model to screen 240 compounds from an anti-tuberculosis hit library for their *in vivo* activity and identified 14 highly active compounds. One of the most active compounds was the tetracyclic compound TBA161, which was studied in more detail. Analysis of resistant mutants revealed point mutations in *aspS* (*rv2572c*), encoding an aspartyl-tRNA synthetase. The target was genetically confirmed, and molecular docking studies propose the possible binding of TBA161 in a pocket adjacent to the catalytic site. This study shows that the zebrafish infection model is suitable for rapidly identifying promising scaffolds with *in vivo* activity.

## Abstract (precis)

**Summary:** Exploitation of the medium-throughput capabilities of a zebrafish embryo infection model of tuberculosis to screen compounds for their *in vivo* activity, one of which was characterized as an aspartyl-tRNA synthetase inhibitor.

## INTRODUCTION

The disease tuberculosis (TB), caused by *Mycobacterium tuberculosis* (Mtb), is the deadliest bacterial infectious disease and is responsible for more than 1.5 million deaths annually (World Health Organization, 2020a). Owing to the emergence and increasing rate of multi and extensively drug-resistant strains, there is an urgent need to develop novel anti-TB drugs (Khawbung et al., 2021). Although drug discovery efforts have recently increased (Stop TB Partnership, 2019), the preclinical bottlenecks, such as *in vivo* efficacy testing, have limited the number of compounds reaching clinical studies (Koul et al., 2011).

Numerous whole-cell based drug screening campaigns have yielded an extensive set of anti-mycobacterial compounds that are active *in vitro* against growing Mtb (Ananthan et al., 2009; Ballell et al., 2013; Maddry et al., 2009; Ollinger et al., 2019; Pethe et al., 2010). However, often promising compounds fail during *in vivo* studies due to unexpected toxicity and lack of *in vivo* efficacy (Ekins et al., 2014; Mukhopadhyay and Peterson, 2006; Pethe et al., 2010). The lack of efficacy is potentially attributed to the unfavorable ADME (adsorption, distribution, metabolism and excretion) properties of the compound that cannot be sufficiently assessed during *in vitro* screens (Mukhopadhyay and Peterson, 2006; Pethe et al., 2010). *In vivo* studies that use traditional models, such as rodents or primates, are expensive, space and time consuming, labor intensive and ethically questionable as high-throughput screening (HTS) models (Mukhopadhyay and Peterson, 2006; Singh and Gupta, 2018). Consequently, there is a need for alternative screening strategies to predict the safety and efficacy of drugs in mammalian models.

Previously, an early life-stage infection model of *Mycobacterium marinum* and zebrafish (*Danio rerio*) embryos was proposed to evaluate anti-TB compounds (Dalton et al., 2017; Ordas et al., 2015; Takaki et al., 2012). *M. marinum* is a close genetic relative of Mtb and is advantageous due to its shorter replication time and lower safety regulations (biosafety level 2 organism) (Tobin and Ramakrishnan, 2008). Although *M. marinum* causes opportunistic skin infection in humans, it is a natural pathogen of ectothermic animals, such as zebrafish, and causes TB-like disease (Jernigan and Farr, 2000). Additional advantages of using zebrafish embryos are their high fecundity, rapid development and limited ethical constraints up to 120 h post-fertilization (hpf) (Cronan and Tobin, 2014). Moreover, due to the optical transparency of zebrafish embryos, infection progress can be easily followed in real time (Davis et al., 2002). A steadily increasing amount of studies have proven and validated the *M. marinum*-zebrafish infection model for efficiently modeling mycobacterial pathogenesis (Lesley and Ramakrishnan, 2008; Prouty et al., 2003; Van Der Sar et al., 2004) and the innate immune response of the host (Benard et al., 2016; Meijer et al., 2005; Van Der Vaart et al., 2012). Notably, the formation of hypoxic and necrotic granulomatous lesions has been reported in infected zebrafish (Davis et al., 2002; Stoop et al., 2011), which is one of the hallmarks of human infection with Mtb.

Furthermore, zebrafish embryos were used to evaluate the efficacy and toxicity of several anti-TB drugs (Ho et al., 2021; Makarov et al., 2014; Ordas et al., 2015), including PBTZ169 (macozinone), which is currently in phase 2 of a clinical trial. The *M. marinum*-zebrafish model can be established by injecting bacteria via the caudal vein or by injection in the yolk. Caudal vein injections require precision and are therefore performed manually, which is a labor-intensive endeavor allowing for hundreds of injections per day (Davis et al., 2002). Conversely, yolk injections can be performed with an automated robotic system, resulting in 1000 infected embryos per hour (Carvalho et al., 2011; Ordas et al., 2015; Veneman et al., 2014; Wang et al., 2007). Furthermore, we show that the waterborne treatment of infected embryos allows the selection of active compounds that are absorbable through the zebrafish skin, which we determined to correlate with the oral uptake of antibiotics in humans. This is an important consideration as oral bioavailability is an essential prerequisite for novel anti-TB drugs, aiming to improve the current TB treatment regimens (World Health Organization, 2020b).

In the present study, we optimized the previously described robotic yolk injection procedure in zebrafish (Ordas et al., 2015) to achieve higher throughput with the same reliability. We further used the platform to rapidly screen and identify anti-mycobacterial compounds and scaffolds that show excellent *in vivo* activity. Among our hits, we characterized a novel compound targeting the mycobacterial aspartyl-tRNA synthetase (AspS).

## RESULTS

### Developing a medium-throughput *in vivo* screen

**Fig. 1.**
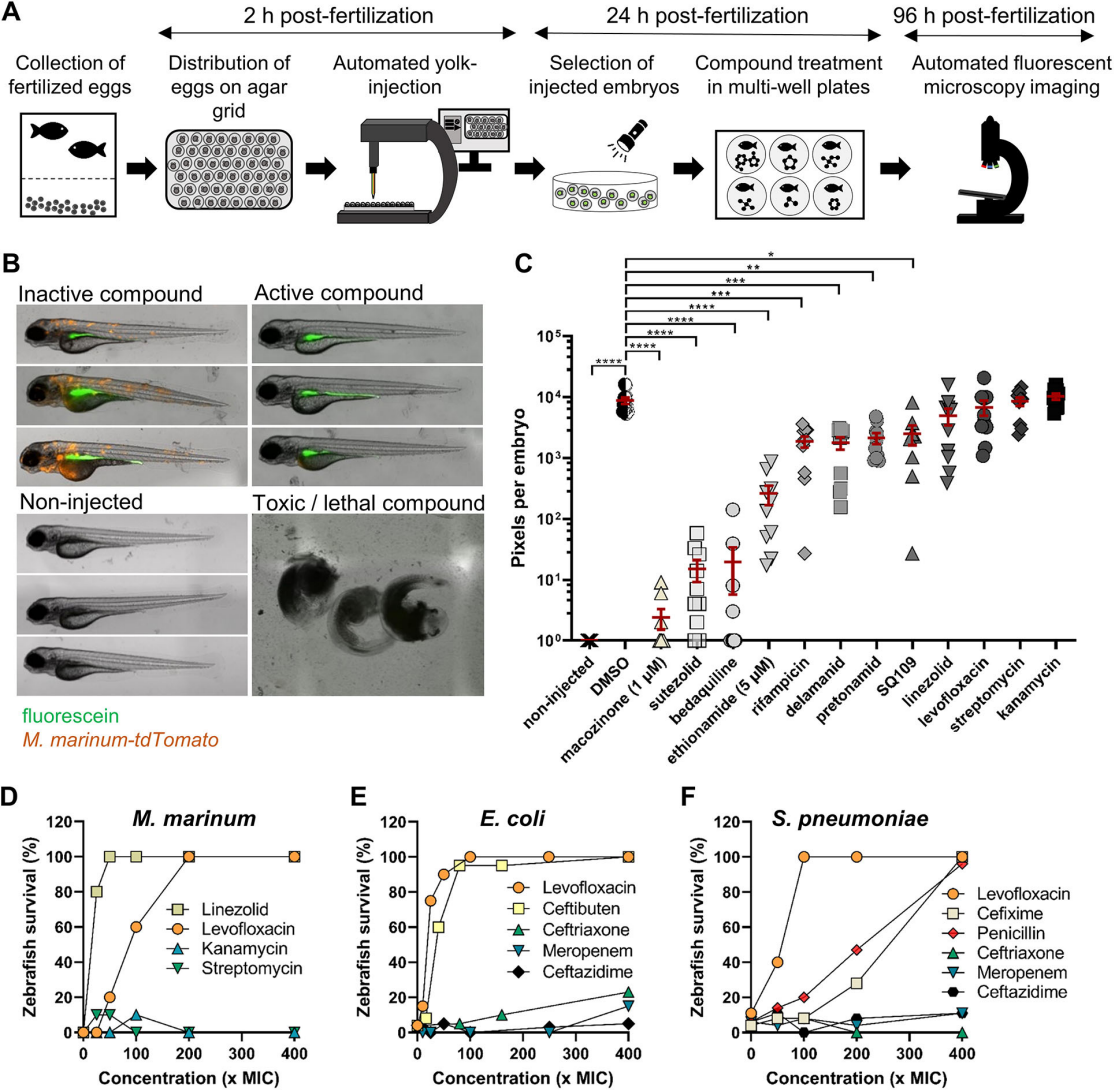
**A zebrafish embryo infection model can be used for medium-throughput compound screening and can predict the oral bioavailability of test compounds.** (A) Schematic representation of the *in vivo* drug-screening setup in the zebrafish-*M. marinum* infection model. (B) Representative images of different readout groups of *M. marinum*-infected zebrafish embryos. (C) *M. marinum*-*tdTomato* yolk-infected zebrafish embryos treated with antibiotics at 10 µM, or as specified. Each data point represents the integrated red fluorescence intensity of a single zebrafish embryo, and the signal of each group (10-12 embryos) is expressed as mean±s.e.m. Statistical significance was determined by one-way ANOVA, following Dunnett's multiple comparison test by comparing the signal from the DMSO-treated control sample with each treatment group (*P≤0.05, **P≤0.01, ***P≤0.001, ****P≤0.0001). (D) Zebrafish embryos were yolk infected with *M. marinum*-*tdTomato* and at 24 hpi treated by adding the antibiotics into the fish water. Survival was scored 4 dpi. Each group consisted of ten embryos. A non-treated group of embryos (0×MIC) served as control. (E,F) Zebrafish were infected via the caudal vein route with *E. coli* GK1161434 (E) or *S. pneumonia* D39V (F) and treated by the addition of the antibiotics to the fish water at 1 hpi. Survival was scored 24 hpt. Each group consisted of 20-40 embryos. Concentrations of all antibiotics were based on the MIC value of the antibiotic for each strain (see Table S1). A non-treated group of embryos (0×MIC) served as control.

Previous studies have established the automated yolk injection procedure in zebrafish embryos using a robotic system (Carvalho et al., 2011; Ordas et al., 2015; Wang et al., 2007). In our study, we aimed to optimize the protocol to conduct a medium-throughput screen (MTS) of anti-mycobacterial compounds (Fig. 1A). We used an automated robotic micro-injector to inject fluorescently labeled *M. marinum* into the yolk of fertilized zebrafish embryos. Although the robotic yolk injection is fairly accurate, not all embryos are successfully injected. In order to efficiently select for correctly injected embryos, we mixed the bacterial suspension with the green fluorescent dye fluorescein and injected the mixture into the zebrafish yolk. Fluorescein allowed for the visualization of the injection procedure in real time and rapid selection of injected embryos based on the green fluorescent signal. The signal of fluorescein did not interfere with the red fluorescent signal that represents the bacterial load (Fig. S1A), and only green-positive embryos were subjected to analysis.

Next, we examined how different infection time-points affect bacterial localization within the zebrafish. When embryos were infected at the 2- to 32-cellular stage, the bacterial aggregates were detected in the yolk, head, tail and body of the zebrafish, which is also observed after the caudal vein infection (Fig. S1B) and is established in the field to represent early granulomas (Davis et al., 2002; Stoop et al., 2011). Conversely, yolk infection at the 64- to 512-cellular stage resulted in bacterial accumulation exclusively in the yolk (Fig. S1B). Consequently, to achieve systemic infection, the zebrafish yolk infection was consistently performed no later than the 32-cellular stage.

Injection of ∼100-150 colony-forming units (CFUs) resulted in a peak of infection at 4 days post-infection (dpi), and prolonged incubation resulted in the death of infected embryos. As our goal was to quantify the infection levels per embryo, the treatment readout was at 4 dpi. Additionally, this timepoint allowed us to discriminate between toxic and non-toxic compounds based on the early lethality or phenotypical changes of embryos in each treatment group (Fig. 1B).

### The zebrafish infection model can predict the oral bioavailability of tested compounds

To validate our optimized *in vivo* screening approach, we tested several anti-TB drugs that are currently available or in clinical trials. Treatment of infected embryos was performed by adding the drugs directly into the fish water. This drug administration route is straightforward and highly suitable for MTS. All drugs were tested at a single concentration of 10 µM, except for macozinone (1 µM) and ethionamide (5 µM), which were, due to toxicity, tested at lower concentrations. On 3 dpi, the bacterial load in each embryo was quantified using integrated red-fluorescent pixel intensity as a readout. Treatment with macozinone, sutezolid, bedaquiline, ethionamide, rifampicin, delamanid, pretonamid and SQ109 resulted in a significant reduction of the bacterial signal (Fig. 1C). However, treatment with the approved anti-TB drugs streptomycin, kanamycin, linezolid and levofloxacin at 10 µM did not reduce the bacterial load in infected embryos. The infection levels were comparable to the levels of control treatment with the solvent DMSO (Fig. 1C).

Our initial setup for drug testing only determined the antibacterial activity at a constant concentration of 10 µM of an antibiotic, a concentration that is widely used for drug screening campaigns to select for highly active and specific hits. We investigated whether levofloxacin, linezolid, kanamycin and streptomycin would show antibacterial activity if they were tested at higher concentrations in a zebrafish infection survival assay. The treatment of infected zebrafish embryos was based on the minimum inhibitory concentration (MIC) of the antibiotics determined in culture (Table S1). A concentration series from 25× to 400× MIC of the different antibiotics was added to the fish water at 1 dpi, and the survival of the embryos was analyzed at 4 dpi. We observed a dose-dependent increase in zebrafish survival when treated with linezolid or levofloxacin (Fig. 1D).

Conversely, the antibiotics kanamycin and streptomycin showed no activity even at 400× of the MIC value (Fig. 1D). We speculated that the inactivity might be due to poor uptake of the compound into the zebrafish embryo. Hence, we injected the compounds via the caudal vein at 1 dpi and observed a significant decrease in the bacterial load (Fig. S2A). These results demonstrate that streptomycin and kanamycin can reduce the infection in zebrafish, but only when injected directly into the bloodstream. The antibiotics streptomycin and kanamycin are clinically well established and effectively treat TB in patients. However, both antibiotics are administered via intravenous or intramuscular injections; hence, we postulated that the zebrafish model could predict the oral bioavailability of the tested compounds if compounds were administered into the fish water.

To test this hypothesis on a broader scale, we used the zebrafish embryo infection model with Gram-negative bacteria *Escherichia coli* and Gram-positive bacteria *Streptococcus pneumoniae* as infectious agents, and different beta-lactam antibiotics that are either used for oral treatment (ceftibuten, cefixime and penicillin) or intravenous injection (ceftazidime, ceftriaxone and meropenem). We chose this antibiotic class because it consists of several drugs that are comparable in their mode of action and differ mainly in their administration route. As a control treatment, we included the oral drug levofloxacin, a second-generation fluoroquinolone. Zebrafish embryos were infected via caudal vein injection route at 30 hpf with *E. coli* GK1161434 or *S. pneumoniae* D39V. A concentration range from 1× to 400× *in vitro* MIC (Table S1) of the different antibiotics was added to the fish water 1 hpf, and the survival of the embryos was analyzed 24 h post-treatment (hpt). We observed a dose-dependent survival of infected embryos when treated with levofloxacin, ceftibuten, cefixime and penicillin, whereas the non-treated groups showed a survival below 10% for both pathogens (Fig. 1E,F).

Interestingly, incubation with increasing concentrations of ceftazidime, ceftriaxone or meropenem did not increase the survival of the embryos (Fig. 1E,F). As these drugs are clinically administered as intravenous injections, we investigated their curative potential when injected into the zebrafish. The treatment of *S. pneumoniae*-infected embryos by intravenous injections of 1× or 10× MIC of ceftazidime or meropenem resulted in 100% zebrafish survival (Fig. S2B). Treatment with ceftriaxone needed a concentration of 10× the MIC to obtain 90% survival of *S. pneumoniae*-infected embryos (Fig. S2B). Similar results were obtained for ceftriaxone treatment of *E. coli*-infected embryos (Fig. S2C).

Collectively, our results suggest that antibiotics that are clinically administered via intravenous injections show activity only when injected into the zebrafish bloodstream. Conversely, the antibiotics that are clinically administered as oral drugs showed activity when added to the fish water and when injected. Hence, our results suggest that the waterborne treatment of infected zebrafish embryos during a screen selects compounds with an increased chance of having good oral availability.

### Medium-throughput screening of an anti-Mtb library against *M. marinum*-infected zebrafish embryos

**Fig. 2.**
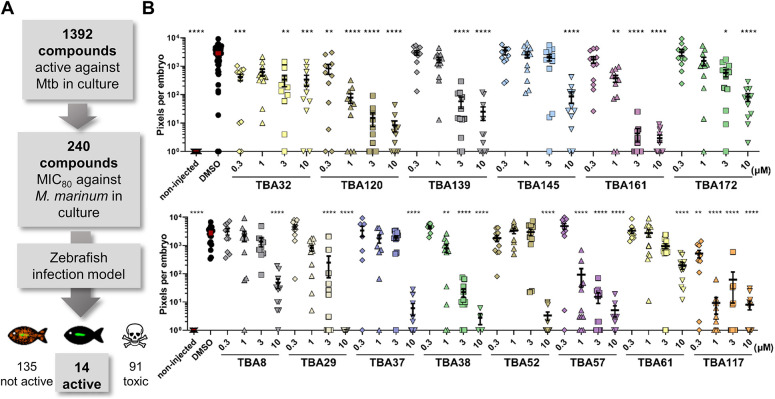
**Screening a library of anti-mycobacterial compounds in zebrafish-infection model identifies 14 hit-compounds.** (A) Schematic representation of the screen design. Compounds active against Mtb and *M. marinum in vitro* were tested in the zebrafish embryo-*M. marinum* yolk-infection model. (B) Hit compounds were tested in a dose-response assay. Each data point represents the integrated red fluorescence intensity of a single zebrafish embryo, and the signal of each group (10-20 embryos) is expressed as mean±s.e.m. Statistical significance was determined by one-way ANOVA, following Dunnett's multiple comparison test by comparing the signal from the DMSO-treated control sample with each treatment group (*P≤0.05, **P≤0.01, ***P≤0.001, ****P≤0.0001).

After the setup of the *in vivo* screening approach, the platform was used to screen compounds from an anti-Mtb hit library [provided by TB Alliance (TBA)] for their *in vivo* activity. This library comprises 1392 compounds previously shown to inhibit Mtb viability *in vitro* (Ho et al., 2021)*.* All compounds that showed ≥80% inhibition of *M. marinum* viability *in vitro* at 10 µM (240 compounds) were selected and tested further in the zebrafish-*M. marinum* infection model via automated yolk injection and waterborne treatment using a single dose (10 µM) (Fig. 2A). From 240 tested compounds, 91 compounds exhibited toxic or lethal activity towards zebrafish embryos at 10 µM and were excluded from further experiments and analysis. Among the 149 non-toxic compounds, we identified 14 compounds that significantly reduced bacterial load in infected zebrafish, the majority in a dose-dependent manner (Fig. 2B). Taken together, only 6% of compounds that were active against *M. marinum* in culture showed significant activity in the early *in vivo* zebrafish infection model, thus highlighting the translational gap between *in vitro* and *in vivo* models.

### *In vivo* activity of compounds cannot be predicted from their physicochemical properties

Our screen showed that the *in vitro* activity of compounds does not translate directly to *in vivo* activity in an infection model. We examined whether *in vivo* activity in the zebrafish infection model could be predicted from the physicochemical properties of the compounds, which for the non-toxic test compounds were collected from online chemical databases (PubChem, ChemInfo; Table S7). The compounds were divided into three groups based on their activity in our model: (1) non-active compounds; (2) active compounds; and (3) active reference antibiotics (macozinone, sutezolid, bedaquiline, ethionamide, rifampicin, delamanid, SQ109 and pretonamid). Next, we performed a principal component analysis (PCA) to investigate whether the correlation between those physicochemical properties can be used as a predictive model for the activity of the compounds. None of the investigated chemical properties or their combinations showed clustering with the activity class of the compounds (Fig. S3A), indicating that the activity of the compounds in the zebrafish infection model cannot be predicted solely on their physicochemical properties. Interestingly, even approved antibiotics did not cluster and showed high variability (Fig. S3A). Although we did not observe clustering of the activity groups of the compounds, the active compounds identified in this study showed more variations in PC2 compared to PC1 (Fig. S3A). We observed that the 14 identified active compounds showed a lower molecular weight and lower complexity compared to all 135 non-active compounds and approved antibiotics investigated in this study (Fig. S3B). The complexity value estimates the complexity of the molecule based on its composing elements and structural features (e.g. symmetry) while excluding the molecular stereochemistry (Bertz, 1981; Hendrickson et al., 1987). However, the current analysis was limited to a large number of inactive compounds compared to a limited number of active compounds. Therefore, we cannot exclude that with more stratifying features and additional active compounds, predictive traits could be extracted.

### Anti-bacterial characterization of 14 hit compounds

We further characterized the 14 hit compounds from the *in vivo* screen by examining their activity in *in vitro* and *ex vivo* assays (Table 1). Compounds were tested against Mtb viability *in vitro*, and all of them showed dose-dependent activity with MIC_50_ values below 10 µM (Table 1). Next, the compounds were tested against Mtb-infected THP-1 macrophages. In this *ex vivo* model, the majority of the compounds showed dose-dependent intracellular activity by reducing the bacterial viability while protecting macrophages from bacterial-induced lysis (Table 1; Fig. S4). However, two compounds, TBA61 and TBA172, did not show activity in this model, whereas they were active against Mtb in culture and in the zebrafish-*M^.^ marinum* infection model (Table 1, Fig. 2B; Fig. S4). Perhaps these compounds are mainly active against extracellular mycobacteria.

**Table 1.**
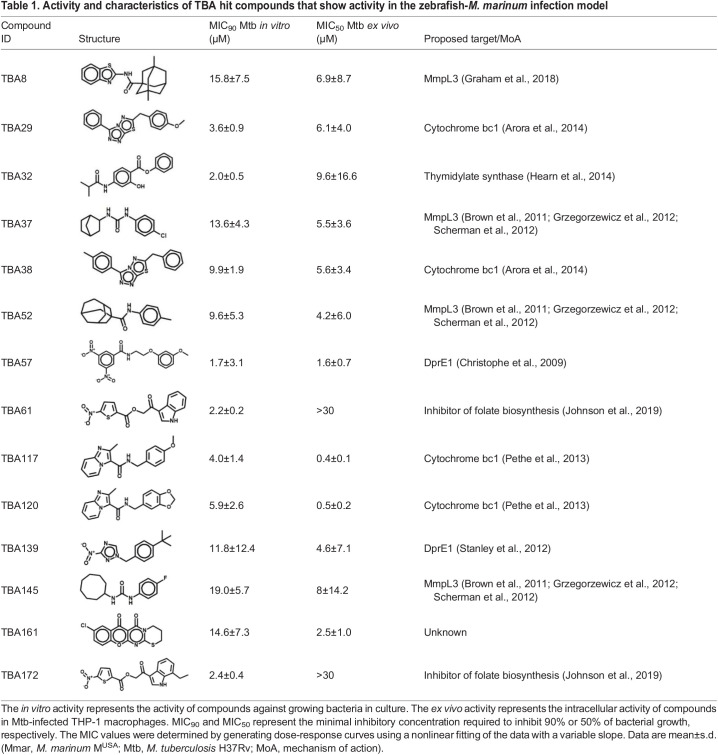
Activity and characteristics of TBA hit compounds that show activity in the zebrafish-*M. marinum* infection model

Most of the identified hit compounds have been described previously in the literature (Table 1). For 13 compounds or their close derivatives, either the target was confirmed or the mechanism of action was proposed based on genetic screens (Table 1). However, the mode of action of hit compound TBA161 was unknown. This compound was among the four most active compounds during fish infection experiments (Fig. 2B). Consequently, we decided to investigate this compound in more detail.

### Characterization of TBA161 variants identified a meta-di-chloro substituted derivative with higher activity

The structure of compound TBA161 consists of four linear fused six-membered substituted heteroatom rings. The two outermost rings are a chloro-substituted benzyl ring and a thiazine on the opposite side of the structure. We obtained four derivatives with alteration in those two ring structures to investigate whether we could identify a more potent compound or find the structural limitations of the compound towards activity (Table 2). The original compound TBA161 is a heterocyclic compound characterized by a single chloride atom on a benzyl ring. TBA161-A consists of a benzyl ring without any substitutes, whereas the derivative TBA161-B differs from the initial compound TBA161 by containing a bromo-benzyl. Compound TBA161-C is characterized by a double substituted meta-di-chlorobenzyl, and in the derivative TBA161-D the thiazine ring was opened.

**Table 2.**
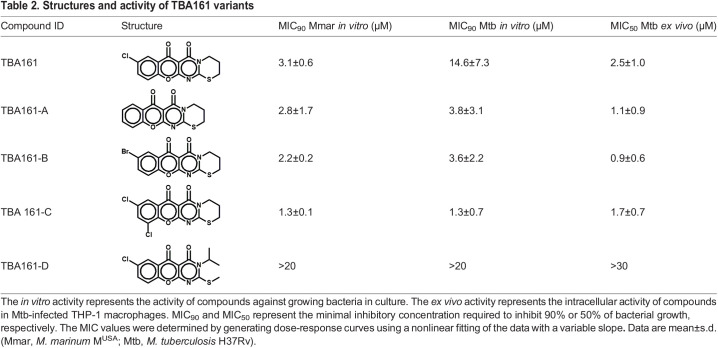
Structures and activity of TBA161 variants

**Fig. 3.**
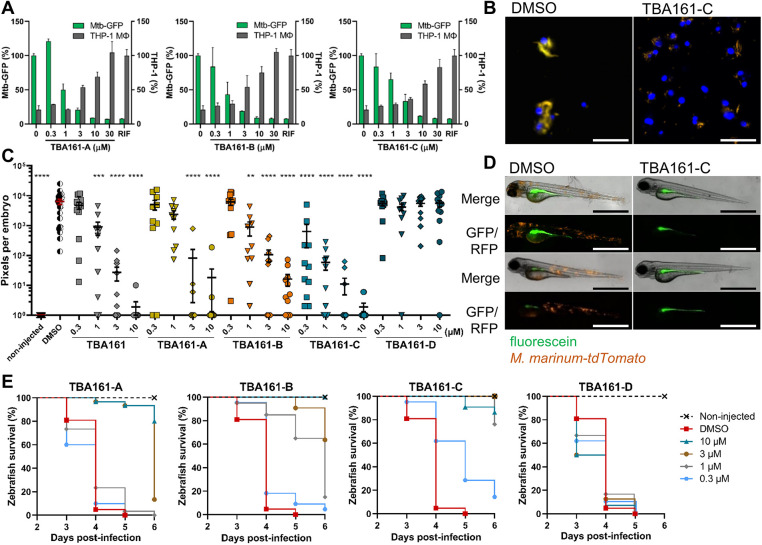
**Activity of TBA161 variants in macrophage and zebrafish infection models.** (A) THP-1 macrophages were infected with Mtb carrying pTetDuo, expressing GFP with the tetracycline-inducible promoter and *tdTomato* under the constitutive promoter p_smyc_. Infected macrophages were treated with various doses of each test compound for 6 days. GFP expression was induced by the addition of ATc, and macrophage nuclei were stained with Hoechst dye to detect macrophages (grey bars). The GFP signal within each macrophage was quantified, representing the amount of viable bacteria (green bars). DMSO- and rifampicin (RIF, 3 µM)-treated samples served as a negative and positive control, respectively. Data points represent the average of duplicates with s.d. (B) Representative images of Mtb-pTetDuo infected THP-1 macrophages treated with DMSO or compound TBA161-C at 6 dpt. Blue, macrophage nuclei (Hoechst); yellow, merged signal of Mtb expressing *tdTomato* (red) and *gfp* (green). Scale bars: 50 µM. (C) Dose-dependent activity of TBA161 variants in the zebrafish-*M.marinum* infection model. Each data point represents the integrated red fluorescence intensity of a single zebrafish embryo, and the signal of each group (10-20 embryos) is expressed as mean±s.e.m. Statistical significance was determined by one-way ANOVA, following Dunnett's multiple comparison test by comparing the signal from the DMSO-treated control sample with each treatment group (**P≤0.01, ***P≤0.001, ****P≤0.0001). (D) Representative images of *M. marinum-tdTomato* yolk-infected zebrafish embryos treated with DMSO (left) or TBA161-C (right) at 3 dpt. (E) Survival curves of *M. marinum* yolk-infected zebrafish embryos after dose-dependent drug treatment. The treatment started at 1 dpi by adding compounds to the fish water. Each treatment group consisted of 25-30 embryos. Scale bars: 1 mm.

All derivatives were tested for their activity against Mtb and *M. marinum* in culture. The compound with the most potent activity against both bacteria was TBA161-C, whereas TBA161-B and TBA161-A showed similar activity to the initial derivative TBA161 (Table 2). Inactivity of TBA161-D indicates that the thiazine ring is crucial for the activity (Table 2). Next, the TBA161 derivatives that showed activity against Mtb in culture were investigated for intracellular activity using Mtb-infected THP-1 macrophages. All compounds inhibited bacterial growth and protected the Mtb-infected macrophages from lysis in a dose-dependent manner (Fig. 3A,B, Table 2).

The compounds were further investigated for their *in vivo* activity in the zebrafish-*M. marinum* infection model. The results were in line with the *in vitro* data, i.e. all derivatives except TBA161-D showed a significant reduction of bacterial load in a dose-dependent manner, with TBA161-C being the most active compound (Fig. 3C,D). This was additionally confirmed during a zebrafish infection survival experiment, in which embryos were yolk infected with a high number of bacteria (1000 CFU), and the treatment efficacy was scored based on the survival of the zebrafish (Fig. 3E). Compound TBA161-C showed the highest protective efficacy among the TBA161 derivatives (Fig. 3E). Consequently, the results are in agreement with previous *in vitro* and *in vivo* data (Fig. 3C,D, Table 2). We can conclude that the opening of the thiazine ring results in a complete loss of *in vitro* and *in vivo* activity, whereas additional substitution of the benzyl ring to a meta-di-chlorobenzyl significantly increases the activity. All further experiments were performed with the most active derivative TBA161-C.

We tested TBA161-C against various bacterial strains to determine its specificity but we only observed activity towards the slow-growing mycobacteria *M. marinum* and Mtb (Table S2). Moreover, TBA161-C was not cytotoxic to THP-1 monocytes and RAW 264.7 macrophages up to 40 µM, and zebrafish embryos up to 100 µM (Table S2), thus confirming selective activity and a favorable safety profile.

### Spontaneous resistant strains of *M. marinum* and Mtb carry mutations in the gene *aspS*

**Fig. 4.**
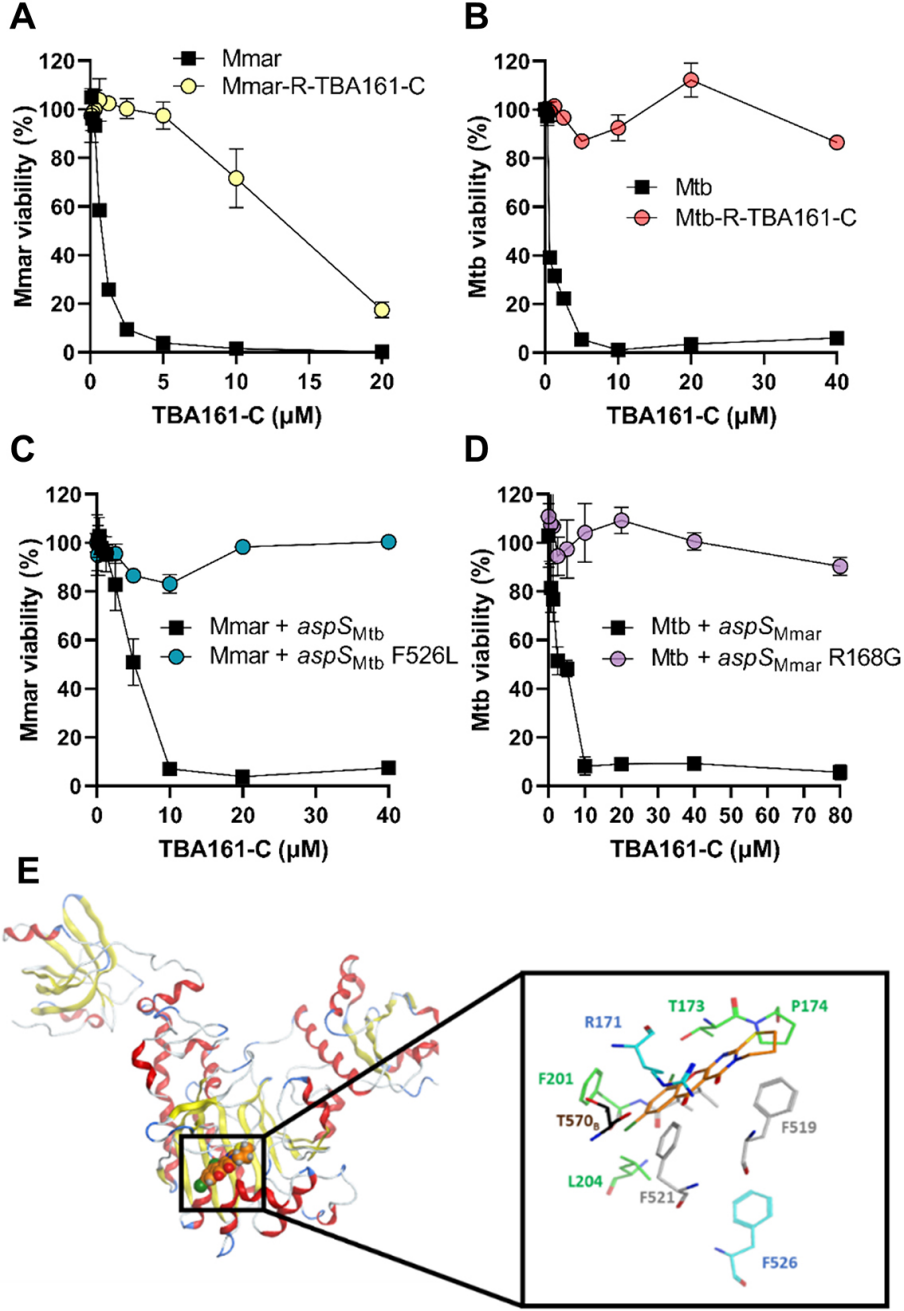
**Mutations in *aspS* are associated with TBA161-C resistance.** (A) Susceptibility of *M. marinum* wild type (Mmar) and TBA161-C-resistant isolates (Mmar-R-TBA161-C) towards TBA161-C after 4 days of incubation. (B) Susceptibility of Mtb wild type and TBA 161-resistant isolates (Mtb-R-TBA161-C) towards TBA161-C was measured after 7 days. (C) *M. marinum* wild type transformed with pMS2-*aspS_Mtb_* (Mmar+*aspS_Mtb_*), and pMS2-*aspS_Mtb_*F526L (Mmar+*aspS_Mtb_*_F526L_) were incubated with compound TBA161-C for 4 days at the indicated concentrations. (D) Mtb carrying plasmids pMS2-*aspS_Mmar_* (Mtb+*aspS_Mmar_)* and pMS2-*aspS_Mmar_*R168G (Mtb+*aspS_Mmar_*_R168G_) were incubated with twofold dilutions of compound TBA161-C for 7 days. (E) TBA161-C (orange) docked into the catalytic subdomain of chain A of Mtb AspS (PDB ID: 5W25). The zoom-in shows TBA161-C in stick representation, together with AspS residues aligning the binding pocket. These include R171 (blue), the three residues of which side chains were treated flexibly during docking (grey), and T570 of chain B (dark brown). The distant F526 residue is shown in blue. For clarity, the L200 label and all hydrogen atoms are omitted. Data are mean of duplicates±s.d.

To identify the target of TBA161-C, we raised spontaneous resistant mutants of Mtb and *M. marinum*. Bacteria were continuously passaged in liquid culture with increasing concentrations of TBA161-C at every passage, resulting in the gradual selection of resistant strains. Single isolates were tested for their susceptibility towards TBA161-C. Both *M. marinum* and Mtb TBA161-C resistant strains showed an MIC_90_ exceeding 20 µM, which is more than tenfold higher compared to the parental strains (Fig. 4A,B). The genomes of three resistant *M. marinum* strains were sequenced and compared to the parental strain. We identified in all resistant isolates the identical two single nucleotide polymorphisms (Table S3A). One of the mutations was located in *mmpL13* (MMAR_4305), resulting in an amino acid substitution P502R. MmpL13 is a conserved transmembrane protein with an unknown function. The second mutation was located in *aspS* (MMAR_2158), causing the amino acid substitution R168G. This gene codes for an aspartyl-tRNA(Asp/Asn) synthetase. According to transposon mutagenesis studies, *mmpL13* is not an essential gene for Mtb or *M. marinum*, whereas *aspS* was shown to be essential in both species (Dejesus et al., 2017; Griffin et al., 2011; Weerdenburg et al., 2015). Thus, we hypothesized that *aspS* might be the molecular target of TBA161-C.

Next, we performed whole-genome sequencing of the TBA161-C resistant Mtb isolate. The analysis revealed mutation in the gene *aspS* (*rv2572c*), resulting in amino acid substitution F526L (Table S3B), indicating that AspS might be involved in TBA161-C resistance in *M. marinum*, as well as in Mtb. In addition, two gene deletions were found (Table S3B): gene *rv0544c*, which encodes for a possible conserved transmembrane protein; and *lprK* (*mce1E* or *rv0173*), a predicted surface lipoprotein. According to the literature, both *lprK* and *rv0544c* are not essential for Mtb growth *in vitro* (Dejesus et al., 2017; Griffin et al., 2011).

As both *M. marinum*- and Mtb-resistant isolates shared mutations in *aspS*, we speculated that *aspS* is the most probable molecular target of TBA161-C. Additionally, we investigated whether *aspS* mutations are also present in other TBA161-C-resistant Mtb isolates. Amplification and sequencing of the gene *aspS* (*rv2572c*) in four resistant Mtb isolates revealed that all strains carried an identical mutation in *aspS*, resulting in amino acid substitution F526L.

### Genetic cross-complementation confirms that TBA161 targets AspS

To confirm the contribution of the identified mutations to TBA161-C resistance, a genetic approach was applied. First, we amplified and cloned wild-type *aspS* and mutated genes of *M. marinum* and Mtb so that they were placed under the control of the constitutive promoter p_smyc_, resulting in four expression vectors. The overexpression vectors encoding the wild-type (*aspS*_Mtb_) and mutated *aspS* (*aspS*_Mtb_F526L) genes of Mtb were transformed into *M. marinum* wild type, and vice versa, Mtb was transformed with the expression constructs of *aspS*_Mmar_ and *aspS*_Mmar_R168G*.* Next, the MIC of all strains against TBA161-C was determined. In both organisms, overexpression of the mutated *aspS* gene caused complete resistance to TBA161-C in *M. marinum* (MIC_90_>40 µM) and in Mtb (MIC_90_>80 µM). Conversely, the strains overexpressing wild-type *aspS* remained susceptible but showed an increased MIC_90_ compared to the wild-type strains (fivefold for *M. marinum* and Mtb) (Fig. 4C,D, Table 2). These results demonstrate that target overexpression itself is not the main cause of resistance.

Nevertheless, we lowered the expression of *aspS*_Mtb_F526L by integrating the same expression cassette into the genome using the mycobacteriophage L5 attachment site and vector pML1342 in *M. marinum*. This integrative vector was previously shown to have 12- to 25-fold lower expression compared to the episomal vectors (Huff et al., 2010). The susceptibility of the strain towards TBA161-C was investigated in an MIC assay. We showed that even when integrated in the genome, the strain expressing *aspS*_Mtb_F526L was resistant against TBA161-C, with an MIC_90_ higher than 40 µM (Fig. S5A).

Next, we investigated whether other mutations found in the TBA161-C resistant isolates could contribute to bacterial resistance towards TBA161-C. The *mmpL* genes have been shown to facilitate the transport of lipids and drugs across the cell envelope (Briffotaux et al., 2017; Grzegorzewicz et al., 2012). To investigate the putative role of *mmpL13* in resistance to TBA161-C, we amplified and cloned *M. marinum* wild-type *mmpL13* and mutated *mmpL13* (P502R) under the control of the constitutive promoter p_smyc_, and transformed the vectors into *M. marinum* wild type. The effect of TBA161-C on *mmpL13*-overexpressing strains was studied in the MIC assay. We found that the overexpression of wild-type or mutated *mmpL13* did not affect the susceptibility of the strains towards TBA161-C (Fig. S5B), thus indicating that TBA161-C resistance in the isolated *M. marinum* mutant is primarily driven through a mutation in *aspS*. At this point, we cannot exclude that the deletions (frameshift mutations) of genes *rv0544c* and *rv0173* in Mtb contribute to resistance to TBA161-C. Future investigations will have to clarify this question. Collectively, our genetic cross-complementation approach confirmed that TBA161-C targets the aspartyl-tRNA(Asp/Asn) synthetase (*aspS*) in *M. marinum* and Mtb. Additionally, the TBA161-C resistant mutant of *M. marinum* displayed cross-resistance to the derivatives TBA161-A and TBA161-B, suggesting that those compounds have the same target (Fig. S6).

### Molecular docking proposes the possible binding of TBA161-C to *aspS*

**Fig. 5.**
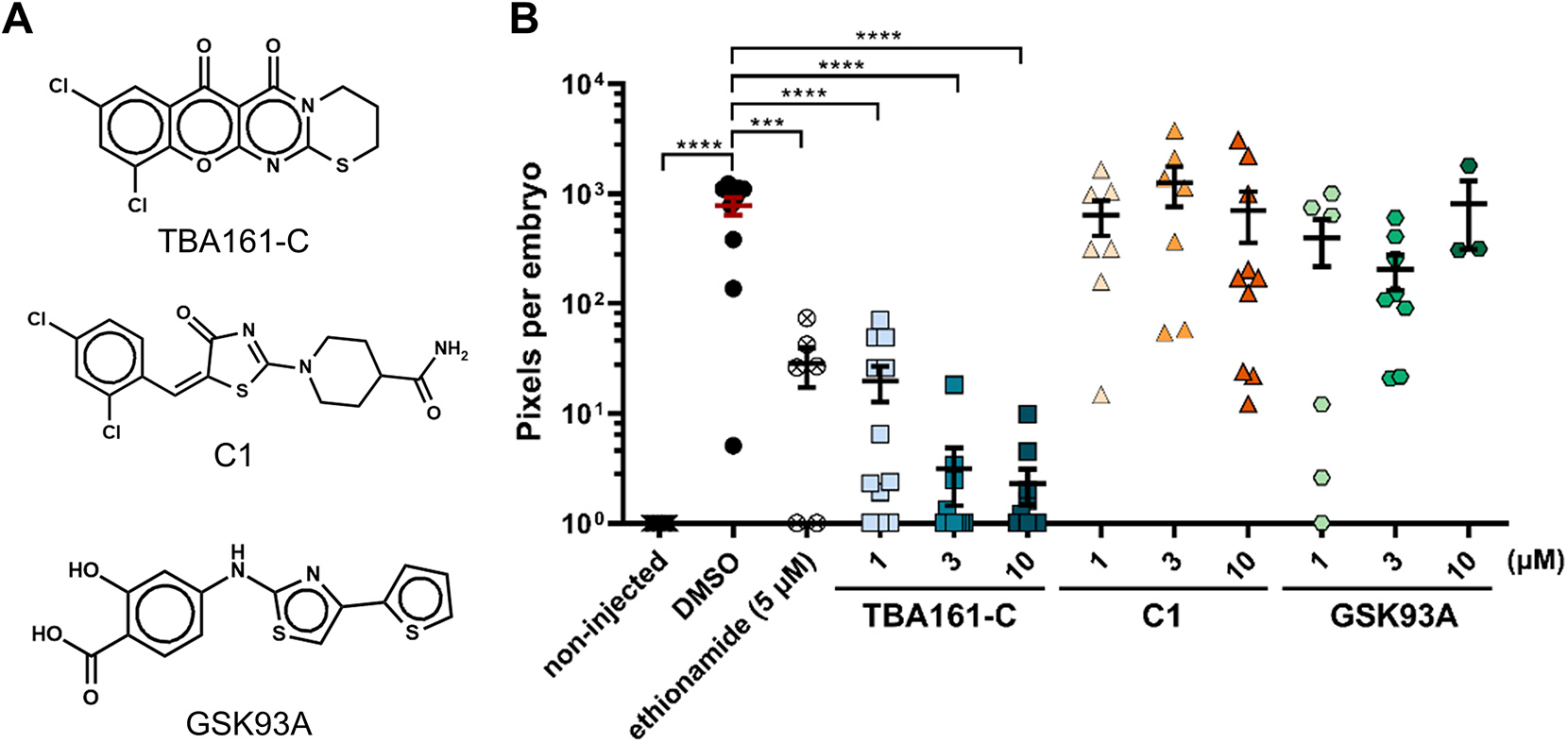
**TBA161-C has potent activity in the zebrafish infection model compared to other AspS inhibitors.** (A) The chemical structures of the test compounds, TBA161-C, C1 and GSK93A. (B) Zebrafish embryos were yolk infected with *M. marinum*-*tdTomato* and treated with compounds at the indicated concentrations. Each data point represents the integrated red fluorescence intensity of a single zebrafish embryo, and the signal of each group is expressed as mean±s.e.m. Statistical significance was determined by one-way ANOVA, following Dunnett's multiple comparison test by comparing the signal from the DMSO-treated control sample with each treatment group (***P≤0.001, ****P≤0.0001).

Using molecular docking, we obtained binding poses of TBA161-C in Mtb *aspS*, in which the central rings of the compound are in direct contact with residue R171 (R168 in *M. marinum*). Fig. 4E shows one of these poses and highlights other Mtb *aspS* residues that are in close vicinity of the compound, including T173, P174, F201, L204 and F519, and T570 of chain B of the 5W25 template structure. These residues correspond to T168, P169, F196, L199, F514 and T565 of *M. smegmatis aspS*, which were previously identified as interacting partners for the compound C1 (Fig. 5A) docked into *M. smegmatis aspS* by Gurcha et al. (2014). One of the chloro-substituents of TBA161-C is found in the apolar cavity formed by L200, F201, L204 and F521, whereas the other may be involved in interactions with the hydroxyl group of the T173 side chain (Fig. 4E). These favorable contacts may occur as a result of the higher activity of TBA161-C compared to the TBA161 derivatives that contain only a single aromatic halogen or lack them altogether. Our docking results do not offer a direct explanation for the lack of activity of TBA161-D compared to the other variants (Table 2). In addition, Fig. 4E depicts residue F526 of Mtb *aspS*, which is at a distance of more than 1 nm from the docked compound and hence does not show interaction with TBA161-C.

### TBA161-C is a potent AspS inhibitor showing excellent *in vivo* activity

Previous studies reported several distinct compounds as AspS inhibitors in mycobacteria. Compound C1 was proposed to be an AspS inhibitor due to the identification of mutations in *aspS* of C1-resistant Mtb strains (Ioerger et al., 2013). Another study identified the inhibitor GSK93A during an *in vitro* whole-cell based screen established to identify AspS inhibitors by screening a hit library (Soto et al., 2018). Here, we compared the activity of these two AspS inhibitors with our newly identified compound TBA161-C. Interestingly, these compounds show diverse chemical structures with only minor similarities, as demonstrated by a Tanimoto coefficient below 0.5 (Fig. 5A; Fig. S7A). First, we compared the *in vitro* activity of the compounds against Mtb. C1 showed comparable activity to TBA161-C, with an MIC_90_ value of 2.5 µM, whereas GSK93A activity was 15-fold lower (Fig. S7B). Compound C1 also inhibited the growth of *M. marinum* in culture, whereas GSK93A did not show activity up to 40 µM (Fig. S7C). Next, we compared TBA161-C, C1 and GSK93A in the zebrafish-*M. marinum* infection model (Fig. 5B). Compound GSK93A was toxic to zebrafish at 10 µM, with only 40% of embryos surviving at 4 days post-fertilization (dpf). Neither GSK93A nor C1 showed activity in the zebrafish infection model, whereas TBA161-C, as we had shown previously, caused a significant reduction of bacterial burden in infected embryos (Fig. 5B). By comparing different compounds with the same proposed target, we can conclude that TBA161-C is a highly active AspS inhibitor *in vivo*, based on our zebrafish embryo infection experiments.

## DISCUSSION

One of the challenges in drug discovery is to rapidly identify compounds that show the most promising characteristics to move on into clinical trials (Koul et al., 2011). As clinical trials are costly, the chances of losing a lead compound in the process should be minimized (DiMasi et al., 2016). To improve the success rate of newly discovered compounds, emphasis has been placed on the predictive value of preclinical model systems (Koul et al., 2011; Wicha et al., 2018).

Widely used HTS platforms allowed large libraries of chemical compounds to be screened on growing bacteria or during macrophage infection studies (Abrahams and Besra, 2020). However, these models do not mimic any of the vital ADME aspects during drug development, which can ultimately lead to a failure of the compound. Therefore, the need to evaluate compounds in animal models remains. However, the HTS of compounds in traditional mammalian models, such as mice, guinea pigs, rabbits and non-human primates, seems nearly impossible (Singh and Gupta, 2018).

Our study aimed to incorporate the *in vivo* model at the earlier steps of the drug discovery route in order to select the compounds with the highest chance to be active in later mammalian models. We used the zebrafish-embryo model, which presents an intriguing middle ground by providing an early *in vivo* infection model for compound evaluation, as well as the ability for medium-throughput screening due to assay miniaturization and process automatization (Bouz and Al Hasawi, 2018; Ordas et al., 2015; Schulthess et al., 2018; Spaink et al., 2013; Takaki et al., 2012). During our study, we screened 240 compounds and showed that only 6% of *in vitro* active compounds exhibited activity in the zebrafish-infection model. Our initial drug screen was based on a single concentration (10 µM) in order to rapidly screen and select highly active compounds. Therefore, the screen is not sensitive enough to identify drugs that are active at higher concentrations, as shown with linezolid and levofloxacin (Fig. 1C,D). Moreover, we tried to establish a mathematical prediction model for the *in vivo* activity of compounds based on their physicochemical properties, but our efforts were unsuccessful. Previous studies reported similar negative results as well (Koul et al., 2011; Lakshminarayana et al., 2015). The low yield of compounds with *in vivo* activity and the inability to predict favorable compounds point to the need for early *in vivo* models.

In the course of our study, we demonstrated that the waterborne treatment of infected fish with antibiotics correlates with the high oral bioavailability of approved antibiotics for humans. However, it remains to be seen how strong the correlation is for the novel hits identified during our screen. Oral drugs are highly desirable for future TB drug regimens (Seung and Hewison, 2019; World Health Organization, 2020b). Currently, all current first-line anti-TB drugs are oral antibiotics (World Health Organization, 2020b). The long treatment times of TB, especially drug-resistant TB, would drastically increase treatment costs and reduce patient compliance, causing once more risk of developing resistant strains if parenteral antibiotics are chosen (Mase and Chorba, 2019; Seung and Hewison, 2019; World Health Organization, 2020b). Thus, a preclinical model with a predictive value for the oral bioavailability of drugs in humans would be highly advantageous. The oral cavity of zebrafish embryos opens at 72 hpf; however, embryos obtain nutrients from the yolk sack up to 7 days post-fertilization (Kimmel et al., 1995). Thus, in the early days, the uptake of drugs by the zebrafish embryo is facilitated exclusively across the zebrafish skin (van Wijk et al., 2019a,b). It has been well established that compounds in adult zebrafish can be taken up across the skin, and these models are extensively used in toxicology and pharmacology (Barros et al., 2008; Cassar et al., 2020; Glover et al., 2013; Morikane et al., 2020; Van Wijk et al., 2019b). It is tempting to speculate that the process of drugs diffusing through the zebrafish skin appears to be similar to the passive uptake of the antibiotics across the human intestinal epithelium. Nevertheless, to verify the specificity of the current screening platform, further validation of the hit compounds and their oral bioavailability is needed.

In order to translate the drug response from zebrafish to humans, the link between internal drug exposure and its response needs to be established (Morgan et al., 2012; van Wijk et al., 2020a). Our screening conditions are very stringent: one-time treatment at a single concentration, meaning that the compound needs to be relatively stable in water and tissue, absorbable in high enough concentrations to reach the target tissue and have sterilizing activity. Besides that, the metabolism of the compound within the zebrafish should be slow. However, one remaining question of our study is the internal drug concentration within the infected zebrafish. Recently, a proof-of-concept study from van Wijk et al. (2020b) showed that the isoniazid concentration in blood was only 20% of the external drug concentration. Using pharmacokinetic-pharmacodynamic modeling, the authors described the exposure-response relationship and concluded that the early bactericidal effect of isoniazid in human infections translates to the responses observed in the zebrafish model (van Wijk et al., 2020b). A similar study was performed on paracetamol, showing that blood concentrations in the embryo were only 10% of the external paracetamol concentration in the fish water (Van Wijk et al., 2019b). Furthermore, after determining the pharmacokinetic properties, the authors showed that absorption, distribution and elimination correlate well with parameters found in higher vertebrates, including humans (Kantae et al., 2016; Van Wijk et al., 2019b). Overall, based on previous reports and an increase in the understanding of the translational value of the zebrafish infection model, we believe that compounds that exhibit activity in the zebrafish infection model have a great potential to be effective in mammalian infection models. Notably, some of the compounds identified as the 14 hit compounds during our screen in the zebrafish infection model were previously reported to show activity in a mouse infection model: a variant of TBA57 is active against Mtb *in vivo* (Christophe et al., 2009); an optimized version of compound TBA8 was shown to be active against *M. abscessus in vivo* (De Groote et al., 2018); and TBA117 and TBA120 are both related to Telacebec (Q203). Telacebec has successfully completed phase 2 clinical trials to be developed as an oral TB-drug (de Jager et al., 2020).

One of the most active compounds in our study during zebrafish and macrophage infection experiments was TBA161. In this study, we identified and confirmed the aspartyl-tRNA synthetase (AspS or AspRS) to be the molecular target of this scaffold in mycobacteria. AspS is a class II aminoacyl-tRNA synthetase (aaRSs; Guo and Schimmel, 2012). These are essential enzymes for protein synthesis as they ligate the specific tRNA molecules to their designated amino acid (Ling et al., 2009). Generally, each aaRS enzyme recognizes a specific amino acid/tRNA pair. However, some prokaryotes, including mycobacteria, do not encode asparagine tRNA synthetase (AsnRS) and glutamine tRNA synthetase (GlnRS) (Paravisi et al., 2009; Woese et al., 2000). Instead, they possess non-discriminating AspS and Glutamyl tRNA synthetase (GluRS) that can, besides Asp/tRNA^Asp^ and Glu/tRNA^Glu,^ also catalyze the formation of Asn/tRNA^Asn^ and Gln/tRNA^Gln^ pairs, respectively (Paravisi et al., 2009; Woese et al., 2000). The aaRSs enzymes represent attractive drug targets (Agarwal and Nair, 2012; Hurdle et al., 2005) as several natural and synthetic compounds have been reported to inhibit different aaRSs (Hurdle et al., 2005). For example, microcin C (McC) and tobramycin were reported as natural products that inhibit bacterial AspS (Walter et al., 2002), and several synthetic compounds have been identified as AspS inhibitors in mycobacteria (Gurcha et al., 2014; Ioerger et al., 2013; Soto et al., 2018). The best-characterized inhibitor, compound C1, was identified by analysis of mutations in *aspS* of C1-resistant Mtb strains (Ioerger et al., 2013). Although C1 and TBA161 are structurally very different, with Tanimoto coefficients below 0.5, we identified that one out of two mutated residues (F526) was identical in AspS_Mtb_ (Ioerger et al., 2013). The outcomes of our docking studies showed that the residue F526 does not play a direct role in inhibitor binding, which was also demonstrated by Gurcha et al. for compound C1. The authors were able to show that in an adjacent subunit of AspS, residue T570 of AspS_Mtb_ can contribute to the binding of compound C1 (Gurcha et al., 2014), which we found for TBA161-C as well. Gurcha et al. reasoned that mutation F526 could cause small conformational changes, which can ultimately weaken the interaction with AspS_Mtb_ residues that are in direct contact with the binding compound (Gurcha et al., 2014). The same effect could contribute to a lower affinity of AspS_F526L_ to TBA161-C and, therefore, resistance. Interestingly, the other residue that contributed to TBA161-C resistance, R171 (R168_Mar_), is located in the binding pocket of AspS and can directly participate in the binding of the inhibitor, as confirmed by docking. So far, no AspS mutation has been associated with the drug resistance in the TB clinical samples (Flandrois et al., 2014; Joshi et al., 2014). The general database of variants detected in TB clinical isolates reports about five AspS mutations (Joshi et al., 2014). Notably, these mutations are distinct from the ones associated with C1 or TBA161-C resistance. Thus, TBA161-C has the potential to become a clinically relevant drug.

Taken together, we identified an anti-mycobacterial inhibitor that targets the essential enzyme AspS within the protein translation pathway and shows excellent activity in the zebrafish infection model. Therefore, the TBA161 scaffold has a high potential for a new drug against Mtb*.* Moreover, this study demonstrates the importance of incorporating early *in vivo* models in the drug discovery pipeline, which will not only accelerate the drug discovery route but also increase its success, saving great costs and efforts.

## MATERIALS AND METHODS

### Bacterial strains, eukaryotic cell lines and culture conditions

All bacterial strains used in this study are listed in Table S4. *M. marinum* M^USA^ was routinely cultured at 30°C in Middlebrook 7H9 medium or on 7H10 agar (Difco) supplemented with 10% ADS (0.5% bovine serum albumin, 0.2% dextrose and 0.085% sodium chloride) and 0.02% tyloxapol. Mtb H37Rv and *Mycobacterium abscessus* were grown in the same medium at 37°C. *E. coli*, *Bacillus subtilis*, *Klebsiella pneumoniae* and *Acinetobacter baumannii* were cultured at 37°C in Luria–Bertani medium (LB; Difco) or on LB agar plates, supplemented with hygromycin (50 μg/ml) where appropriate. *S. pneumoniae* was grown at 37°C with 5% CO_2_ in Todd–Hewitt Broth (Bacto) supplemented with 2% yeast extract (THY; Oxoid), or on Columbia agar plates with 5% sheep blood (COS; Biomerieux) supplemented with chloramphenicol (4.5 μg/ml) where appropriate. THP-1 human monocytes [American Type Culture Collection (ATCC), TIB-202] were routinely cultured in RPMI medium with GlutaMAX (Gibco) supplemented with 10% fetal bovine serum (FBS) at 37°C with 5% CO_2_. RAW 264.7 murine macrophages (ATCC, TIB-71) were routinely cultured in Dulbecco's modified Eagle's medium with GlutaMAX (DMEM; Gibco) supplemented with 10% FBS at 37°C with 5% CO_2_. Both cell lines were obtained from the ATCC and were passaged five times before a fresh culture was started. The tissue cultures were routinely controlled for mycoplasma contamination over 3-6 months using a commercial kit based on PCR.

### Chemical reagents and compound library

Ceftibuten, cefixime, ceftazidime (hydrate), ceftriaxone (sodium), ethionamide, gentamycin, kanamycin (sulfate), levofloxacin, meropenem (trihydrate), penicillin (G sodium), rifampicin (all purchased from Sigma-Aldrich), bedaquiline, delamanid, linezolid, macozinone, pretonamid, streptomycin (sulfate salt), sutezolid, SQ109, 9-chloro-3,4-dihydro-chromeno[2′,3′:4,5]pyrimido[2,1-b][1,3]thiazine-6,7-dione (TBA161) and its derivatives 3,4-dihydro-chromeno[2′,3′:4,5]pyrimido[2,1-b][1,3]thiazine-6,7-dione (TBA161-A), 9-bromo-3,4-dihydro-chromeno[2′,3′:4,5]pyrimido[2,1-b][1,3]thiazine-6,7-dione (TBA161-B), 9,11-dichloro-3,4-dihydro-chromeno[2′,3′:4,5]pyrimido[2,1-b][1,3]thiazine-6,7-dione (TBA161-C) and 7-chloro-3-isopropyl-2-(methylsulfanyl)-chromeno[2,3-d]pyrimidine-4,5-dione (TBA161-D) were all purchased from MedChemExpress, and solubilized and stored according to the manufacturers' recommendations.

The TB Alliance compound library was a gift from TBAlliance (New York, USA). The library consists of 1392 compounds that were previously shown to inhibit Mtb H37Rv viability *in vitro*. Compounds of this library were stored as stock solutions (10 mM) in DMSO at −80°C.

### Construction of plasmids and strains

All primers and plasmids used within this study can be found in Tables S5 and S6, respectively. Plasmids in this study were constructed using standard molecular cloning techniques summarized in Fig. S8.

### Bacterial susceptibility assays

MICs against *Mycobacterium* species were determined using a resazurin reduction microplate assay (REMA), as previously described (Palomino et al., 2002). Briefly, selected compounds or antibiotics were twofold serial diluted in 96-well plates. Bacterial strains were grown to mid-logarithmic phase, harvested by centrifugation (3000* **g***, 10 min), washed in PBS supplemented with tyloxapol (0.02%), resuspended in growth medium and added to each well at a final OD_600_ of 0.001. The lid of each plate was sealed with Scotch tape and plates were incubated for 4 days at 30°C (*M. marinum*), 6 days at 37°C (Mtb) and 2 days at 37°C (*M. abscessus*). Subsequently, 20 µl of resazurin solution [0.025% (w/v) resazurin sodium salt and 20% Tween 80 (ratio 3:1)] were added to each well. After the color conversion of the dye, bacterial viability was measured as fluorescence intensity using a BioTek plate reader (Synergy H1), with bottom reading mode (excitation/emission, 560 nm/590 nm). When using a bacterial strain with a fluorescent marker (*M. marinum*-*tdTomato*), bacterial viability was either measured as fluorescence intensity of tdTomato signal (excitation/emission, 554 nm/581 nm), or the developed resazurin dye was transferred and analyzed in a new plate after the bacteria were pelleted in the 96-well plates (610 ***g***, 5 min).

MICs against non-*Mycobacterium* species were determined using optical density measurements. After overnight growth, the bacterial cells were freshly diluted in an appropriate medium and grown at 37°C to mid-logarithmic phase. Selected compounds or antibiotics were twofold serial diluted in 96-well plates. Then, the cultures were harvested by centrifugation (3000 ***g***, 10 min), washed in PBS and added to each well at a final OD_600_ of 0.001. Plates were sealed and incubated at 37°C for 12 h with 3 mm continuous linear shaking in a BioTek plate reader, and the bacterial growth was measured at OD_600_ every 15 min.

MICs against *S. pneumoniae* were determined using a REMA assay. Selected compounds or antibiotics were twofold serial diluted in 96-well plates. *S. pneumoniae* was grown in Todd-Hewitt broth supplemented with 2% yeast extract (THY) to mid-logarithmic phase and diluted to an OD_600_ of 0.05 in each well of the 96-well plate. Plates were incubated overnight at 37°C with 5% CO_2_. The following day, 10 µl of 0.025% (w/v) resazurin sodium salt solution was added to each well and plates were incubated for 3 h at 37°C. Next, the fluorescence intensity was measured using a BioTek plate reader, with bottom reading mode (excitation/emission, 560 nm/590 nm).

The data for each 96-well plate were normalized to DMSO-treated wells (100% viability) after background subtraction. All compounds were tested in duplicates.

### Zebrafish maintenance

Zebrafish used in this study were *casper* (*roy^a9/a9^;nac^w2/w2^*) compound homozygote mutant fish that completely lack all melanocytes and iridophores in both embryogenesis and adulthood (White et al., 2008). Adult fish were kept in recirculating tank systems at the Amsterdam Animal Research Center of the Vrije Universiteit University under a 14 h/10 h light/dark cycle at pH 7.5 and 26°C according to standard protocols (zfin.org). Zebrafish care, breeding and experiments were performed in compliance with local animal welfare laws [Animal Experimental Licensing Committee, Dier Experimenten Commissie (DEC)]. All protocols adhered to the international guidelines specified by the EU Animal Protection Directive 86/609/EEC, which allows zebrafish embryos to be used up to the moment of free living.

### Infection of zebrafish

Injection stocks of *M. marinum* M^USA^-*tdTomato* and *E. coli* GK1161434 were prepared in PBS with 20% glycerol, aliquoted and stored at −80°C. For injection of *S. pneumonia* D39V, a fresh culture was used. Before use, the injection stock was diluted 1:1 in PBS containing 0.17% (v/v) Phenol Red (Sigma-Aldrich) or 2.5 µg/ml fluorescein (Sigma-Aldrich) to aid visualization of the injection process. The number of injected bacteria was determined by plating the injection volume of bacterial suspension on appropriate plates, followed by counting CFUs.

#### Yolk infection

Transparent *casper* (White et al., 2008) zebrafish embryos were infected using an automated microinjection system (Life Science Methods BV) described previously (Spaink et al., 2013). Zebrafish embryos were infected 1 hpf at the 2- to 32-cell stage with 80-150 CFU/nl (1000 CFU/nl during survival experiments, Fig. 3E) of *M. marinum* M^USA^*-tdTomato* mixed with fluorescein (2.5 µg/ml in PBS). Successfully infected embryos were selected by the detection of green fluorescence and were incubated overnight at 31°C in E3 medium (5.0 mM NaCl, 0.17 mM KCl, 0.33 mM CaCl*2H_2_O and 0.33 mM MgCl_2_*6H_2_O) supplemented with 0.3 mg/l Methylene Blue until antibiotic treatment.

#### Caudal vein infection

Transparent *casper* (White et al., 2008) zebrafish embryos were collected and incubated in E3 medium supplemented with 0.3 mg/l Methylene Blue. Prior to infection, embryos were mechanically dechorionated and anesthetized in 0.02% (w/v) buffered 3-aminobenzoic acid methyl ester (pH 7.0) (tricaine; Sigma-Aldrich, A5040). The zebrafish embryos were individually infected via the caudal vein route, as described previously (Benard et al., 2012; Jim et al., 2016). Successfully infected embryos were collected and incubated at 28°C in E3 medium supplemented with 0.3 mg/l Methylene Blue until antibiotic treatment.

### Compound treatment of zebrafish

#### Waterborne treatment

At 1 dpi with *M. marinum* M^USA^-*tdTomato* or 1 h post-infection (hpi) with *E. coli* GK1161434 or *S. pneumoniae* D39, embryos were divided into treatment groups of 12-15 embryos per well. Embryos were treated with test compounds diluted in fish water (60 µg/ml instant ocean sea salts) and incubated at 28°C. The survival rate was determined daily based on the functioning of the heart and blood circulation of the embryos.

#### Intravenous injection

At 1 dpi with *M. marinum* M^USA^-*tdTomato* or 1 hpi with *E. coli* GK1161434 or *S. pneumoniae* D39V, zebrafish embryos were re-injected with 1 nl of different antibiotics at indicated concentrations. Infected and intravenously treated zebrafish were incubated in fish water at 28°C. The survival rate was determined daily based on heartbeat and blood circulation.

### Determination of bacterial load in infected zebrafish embryos

Three days after the treatment, the *M. marinum*-*tdTomato*-infected zebrafish were anesthetized in 0.02% (w/v) buffered 3-aminobenzoic acid methyl ester (pH 7.0) (tricaine; Sigma-Aldrich), and the bacterial load was monitored using an Olympus IX83 fluorescence microscope (4× objective magnification, Hamamatsu ORCA-Flash 4.0 camera) at specific wavelengths (excitation/emission, 470 nm/519 nm; 550 nm/610 nm). Obtained images were analyzed using CellProfiler 3.19 (Broad Institute, Cambridge, MA, USA) with a custom-made pipeline to count and quantify pixel intensity within the embryos. Integrated red fluorescence intensity per embryo was used as a readout for bacterial burden. Image acquisition and image analysis were automated.

### Zebrafish toxicity studies

Transparent *casper* (White et al., 2008) zebrafish embryos were collected within the first hours post-fertilization and kept overnight at 31°C in E3 medium supplemented with 0.3 mg/l Methylene Blue. At 1 dpf, zebrafish embryos were treated with compounds diluted in fish water at the indicated concentration. Zebrafish embryos were incubated at 28°C for 5 days, and the morphology and mortality of zebrafish embryos were monitored daily.

### Generation and characterization of spontaneous TBA161-C-resistant mutants

Spontaneously resistant mutants of *M. marinum*-*tdTomato* or Mtb strains were generated using natural selection strategies. TBA161-C-resistant mutants from both strains were isolated from 7H9 cultures over five passages with increasing concentrations of TBA161-C, starting from 0.3×, 1×, 3× and 6× MIC to final concentrations of 10× MIC for *M. marinum* and Mtb. Single colonies were obtained by streaking cultures on 7H10 plates. The resistance to TBA161-C was determined by testing the susceptibility of strains to TBA161-C using a REMA assay. Genomic DNA extraction of TBA161-C-resistant and parental mycobacterial strains was performed using phenol/chloroform/isoamyl-alcohol extraction as described previously (Mve-Obiang et al., 2001). Whole-genome sequencing of genomic DNA from parental wild-type *M. marinum*-*tdTomato*, three TBA161-C-resistant *M. marinum*-*tdTomato* strains and TBA161-C-resistant Mtb strain were outsourced to Beijing Novogene Bioinformatics Technology (Novogene, China) using Illumina sequencing technology. Generated reads were aligned to the reference genome of *M. marinum* M^USA^ (NC_010612.1) or Mtb H37Rv (NC_000962.3) and compared to the parental strain using Qiagen CLC Genomics Workbench 12. Three other TBA161-C-resistant Mtb mutant strains were analyzed by Sanger sequencing after amplification of the *aspS* gene by PCR.

### Cytotoxicity

Compounds were distributed as twofold serial dilutions in RPMI GlutaMAX with 10% FBS and incubated with THP-1 monocytes (2.5×10^4^ cells/well) or in DMEM GlutaMAX with 10% FBS, and incubated with RAW macrophages (2.5×10^4^ cells/well) for 3 days at 37°C with 5% CO_2_. After incubation, resazurin sodium salt [0.0025% (w/v) in PBS] was added to the wells, and plates were incubated for 4 h at 37°C. Cell viability was measured as fluorescent intensity using a BioTek plate reader (excitation/emission, 560 nm/590 nm).

### Assessment of intracellular drug activity in infected THP-1 macrophages

Mtb transformed with pTetDuo was grown in 7H9 at 37°C to mid-logarithmic phase, then harvested and washed in PBS. Bacterial infection stocks were prepared in RPMI GlutaMAX with 10% FBS (infection medium) with 20% glycerol, aliquoted and stored at −80°C. THP-1 human monocytes were seeded into black 96-well plates (Ibidi) as 10^5^ cells/well and incubated with phorbol-12-myristate-13-acetate (25 ng/ml) for 48 h at 37°C with 5% CO_2_ to induce differentiation to macrophage-like cells. Differentiated macrophages were washed in infection medium and then infected with Mtb H37Rv carrying pTetDuo at a multiplicity of infection of 5. After 3 h incubation at 37°C with 5% CO_2_, extracellular bacteria were killed by the addition of gentamycin (50 µg/ml) for 1 h at 37°C with 5% CO_2_. After incubation, the medium was replaced with the test compounds, which were prepared in separate 96-well plates by threefold serial dilutions in infection medium. Plates were incubated for 4 days at 37°C with 5% CO_2_. After incubation, anhydrotetracycline (ATc) solution (100 ng/ml) in RPMI was added and plates were incubated for an additional 24 h at 37°C with 5% CO_2_. The medium was replaced with 160 µl of paraformaldehyde [3.2% (w/v)] in PBS, followed by incubation for 30 min at room temperature. The fixating solution replaced with 160 µl of quenching/staining solution [0.1 M glycine, 0.2% (w/v) Triton X-100 and Hoechst dye 1:500 in PBS] was added and incubated for 1 h in the dark. All wells were washed two times with PBS. An Olympus IX83 fluorescence microscope (20× objective magnification) with a Hamamatsu ORCA-Flash 4.0 camera was used to acquire images of each well at specific wavelengths (excitation/emission, 385 nm/455 nm, 470 nm/519 nm and 550 nm/610 nm). Image analysis was performed using CellProfiler 3.19 with a custom-made pipeline that identifies the macrophages based on the blue Hoechst dye-stained nuclei of the macrophages. To account for the cytosol of the macrophage, the radius around the nuclei (median diameter 8.2 µm) was extended by ten pixels without allowing an overlap with neighboring macrophages (median diameter 11.9 µm). The fluorescent signal of the ATc-inducible GFP was used as a readout for viable intracellular bacteria in each macrophage. The number of stained and detected nuclei was used as a readout for the number of macrophages in each treatment group, and was normalized to the rifampicin-treated (3 µM) sample (100% macrophage viability). The average GFP signal in each treatment group was calculated and normalized on the control DMSO-treated sample (100% bacterial viability).

### Molecular docking

Chain A of crystal structure 5W25 (PDB ID: 5W25) of Mtb *aspS* was protonated using Molecular Operator Environment software (Chemical Computing Group ULC, version 2019.09) and used as a template to dock compound TBA16-C into (after the removal of crystal water molecules). Docking was performed using the Protein-Ligand ANT System (PLANTS, version 1.2) software (Korb et al., 2007) in combination with the ChemPLP scoring function (Korb et al., 2009). A two-step protocol was used to obtain the docking pose presented in this study. First, TBA161-C was docked into the binding pocket corresponding to the one described by Gurcha et al. (2014) in their structural characterization of *M. smegmatis aspS*, which is highly homologous (82%) to Mtb *aspS*. In this initial step, we set the center of docking close to R171 (R168 in *M. marinum*; i.e. at the center of the vector connecting CD1 of F519 with CG of R171) and the docking radius to 0.8 nm. Subsequently, we used the coordinates of one of the thus obtained poses with close contacts to R171 as a starting point to further explore the binding pocket in a second docking run, in which a larger docking radius (1.0 nm) was used and the center of docking was set to the center of the vector connecting CG2 of T173 and HE2 of F456. To allow for an induced fit effect, we adapted the side-chain conformations of residues L200, F519 and F521 prior to this second docking run by changing the CA-CB-CG-CD1 dihedral angles of L200 and F519 from 177.3 to −63.1 degrees and −82.5 to 59.0 degrees, respectively, and by changing the F521 C-CA-CB-CG dihedral angle from −58.9 to −151.1 degrees. The above-mentioned Mtb *aspS* residues R171, T173, F456 and F519 correspond to the *M. smegmatis aspS* residues aligning the binding pocket in which Gurcha et al. successfully docked compound C1 into, using their 4RMF crystal structure as docking template. The residues aligning this pocket are conserved between *aspS* of Mtb and *M. smegmatis*, and show nearly identical backbone and side-chain conformations in both the 5W25 and 4RMF structures. To verify our docking approach, we redocked C1, using our protocol, into the 5W25 structure and obtained a similar binding pose as Gurcha et al.

### Principal component analysis

Exploratory data analysis was conducted on data containing the physicochemical properties of compounds, which were either known for *in vitro* activity against Mtb or *M. marinum*. The properties of compounds used for analysis were as follows: molecular weight, logP, logS, polar surface area, XLogP3-AA, number of H-donors, number of H-acceptors, heavy atom number, number of rotatable bonds, complexity, number of NO_2_ groups and number of S-atoms (Table S7). For imputation of missing values, data were assumed to be missing at random and were imputed using predictive mean matching from the MICE package using the R statistical programming language (van Buuren and Groothuis-Oudshoorn, 2011). The resulting imputed frame was further *z*-scored and used for PCA.

### Statistical analysis

All statistical analyses in this study were performed using Prism version 9.0.0 (GraphPad, San Diego, CA, USA). The MIC_50_ values represented 50% growth inhibition and MIC_90_ values represented 90% growth inhibition. The MIC values were determined by generating dose-response curves using a nonlinear fitting of the data with a variable slope. The IC_50_ (the half-maximal inhibitory concentration) and TD_50_ (median toxic dose) values were calculated using the same equation.

The effect of drug treatment in infected zebrafish embryos was analyzed using integrated red fluorescence intensity as a readout. Each data point represents a signal from a single zebrafish embryo and each treatment group consisted of a minimum of ten embryos. Embryos with a fluorescent intensity equal to 0 were set to 1 to allow log_10_ transformation. Log_10_ transformation was performed to achieve normal distribution. Further statistical analysis on log_10_ transformed values was performed using a one-way ANOVA, following Dunnett's multiple comparison test by comparing the signal from the DMSO-treated control sample with each treatment group. Significance is indicated as *P≤0.05, **P≤0.01, ***P≤0.001 and ****P≤0.0001.

## Supplementary Material

Supplementary information
