# Supplementary material for: An anti-tuberculosis compound screen using a zebrafish infection model identifies an aspartyl-tRNA synthetase inhibitor
Source: Dis Model Mech. 2021 Dec 23;14(12):dmm049145. doi: 10.1242/dmm.049145 (PMC8713996; doi:10.1242/dmm.049145)
Supplement: Supplementary information [file dmm-14-049145-s1.pdf]

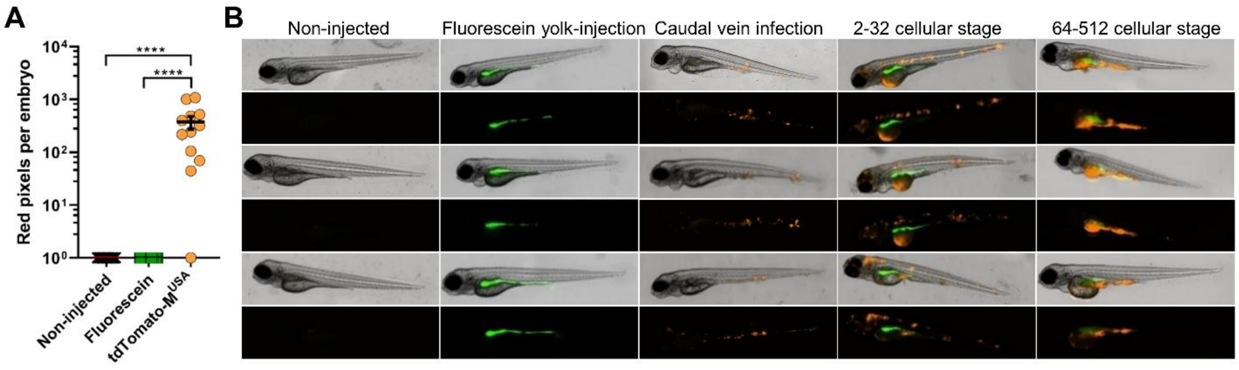

Fig. S1. Bacterial localization within infected zebrafish depends on the time-point of infection. (A) Zebrafish embryos were yolk-infected with fluorescein or *M. marinum* expressing *tdTomato* mixed with fluorescein. At 4 dpi the red-fluorescence intensity was quantified after imaging and used as a readout. Each data point represents the integrated red-fluorescence intensity of a single zebrafish embryo and the signal of each group is expressed as mean ± SEM. Data analysis was performed as described in the methods section. Significance is indicated as: **** $p \leq 0.0001$. **(B)** Representative fluorescence images of embryos infected via caudal vein at 1 dpf or infected via yolk injections at 2-32 or 64-512 cellular stages. Four days after injection, fluorescence images were generated. The green-fluorescent dye fluorescein served as an injection control and the red-fluorescent signal corresponds to the bacterial load of *M. marinum*.

Disease Models & Mechanisms • Supplementary information

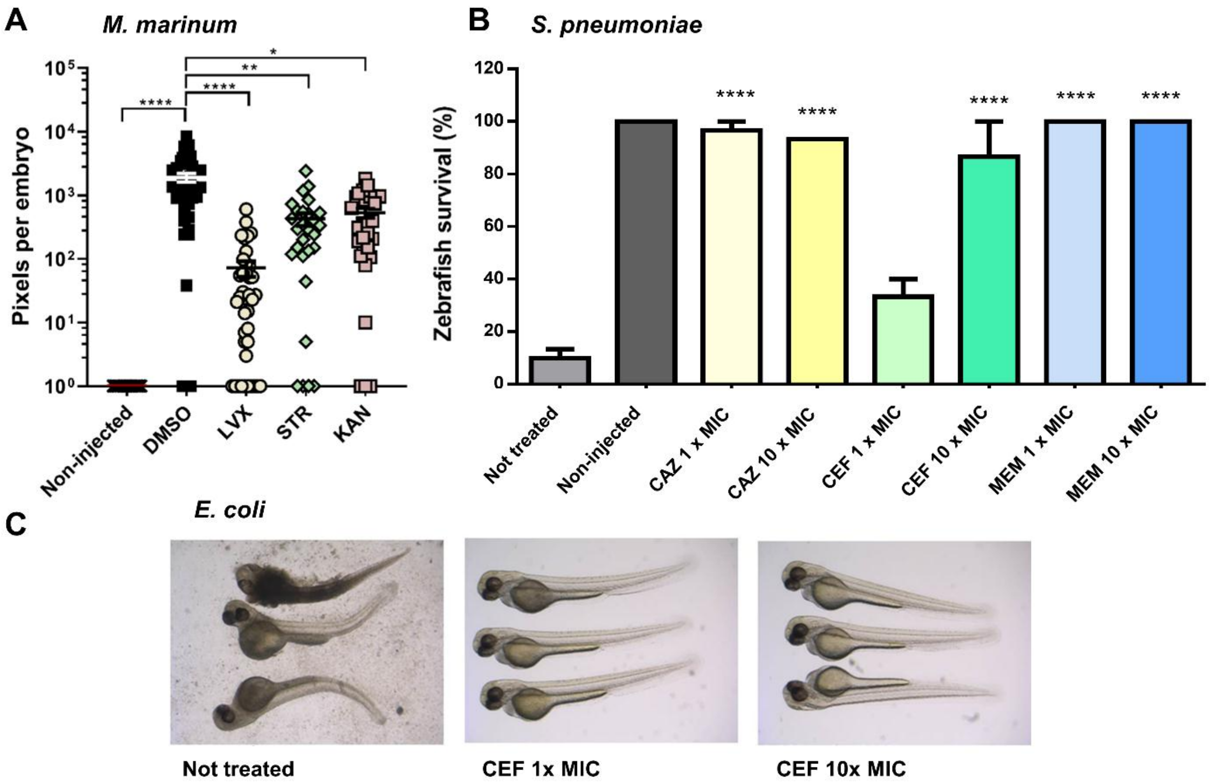

Fig. S2. Treatment of infected zebrafish by intravenous drug injection is effective for antibiotics that are administered intravenously. (A) Zebrafish embryos were 1 dpf intravenously infected with *M. marinum-tdTomato* and 24h later treated by intravenous injection of indicated antibiotics. On 4 dpi fluorescence images were generated and the integrated red-fluorescence intensity was quantified. Each data point represents the integrated red-fluorescence intensity of a single zebrafish embryo and the signal of each group is expressed as mean ± SEM. Data analysis was performed as described in the methods section. Significance is indicated as: * $p \leq 0.05$; ** $p \leq 0.01$; *** $p \leq 0.001$; **** $p \leq 0.0001$. **(B)** Zebrafish embryos were 1 dpf intravenously infected with *S. pneumonia* D39V and 1 hpi treated by intravenous injection with 1 or 10 times the MIC value (see Table S1) of ceftazidime (CAZ), ceftriaxone (CEF), or meropenem (MEM). Infected zebrafish with no treatment and non-infected zebrafish served as negative and positive control, respectively. The zebrafish survival was scored 24 hpt. The significant difference in comparison to the negative control group is indicted as : * $p \leq 0.05$; ** $p \leq 0.01$; *** $p \leq 0.001$; **** $p \leq 0.0001$. **(C)** Zebrafish embryos that were 1 dpf

Disease Models & Mechanisms • Supplementary information

intravenously infected with *E.coli* GSK1161343 and 1 hpf treated by intravenous injection with 1 or 10 times the MIC value (see Table S1) of ceftriaxone (CEF). The control group consisted of non-infected and non-treated zebrafish. The zebrafish survival was scored 24 hpt and is presented as a representative image of each treatment group.

Disease Models & Mechanisms • Supplementary information

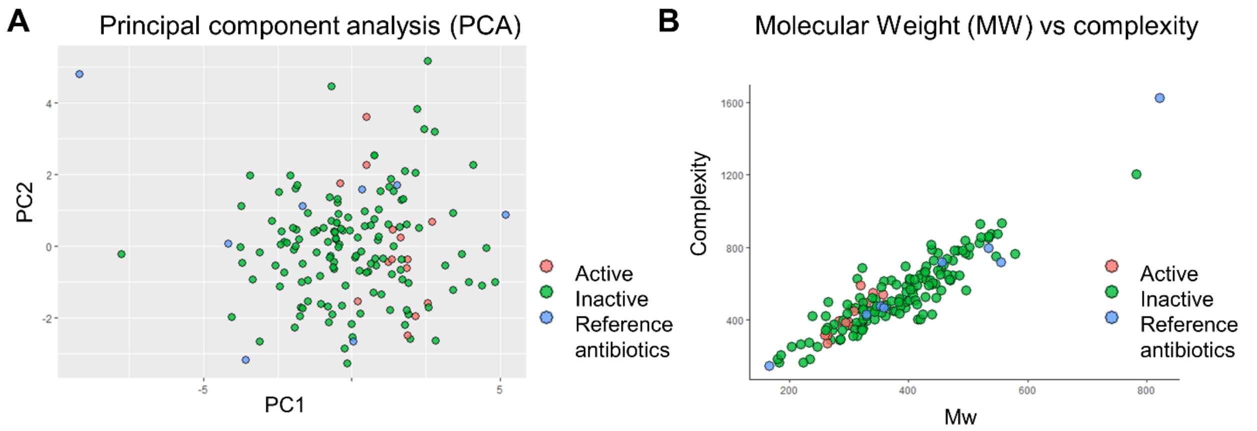

Fig. S3. Compounds activity in the zebrafish infection model cannot be predicted on compounds physicochemical properties. (A) Visual representation of performed principal component analysis (PCA). Compounds were divided into three activity classes, based on their activity in the zebrafish-infection model: active compounds (14), inactive compounds (135), active reference antibiotics (macozinone, sutezolid, bedaquiline, ethionamide, rifampicin, delamanid, SQ109, pretonamid). **(B)** Graph comparing the molecular weight (MW) and complexity of each compound of the three activity classes. Complexity value is based on the elements and structural features of the molecule (Bertz, 2002; Hendrickson et al., 1987).

Disease Models & Mechanisms • Supplementary information

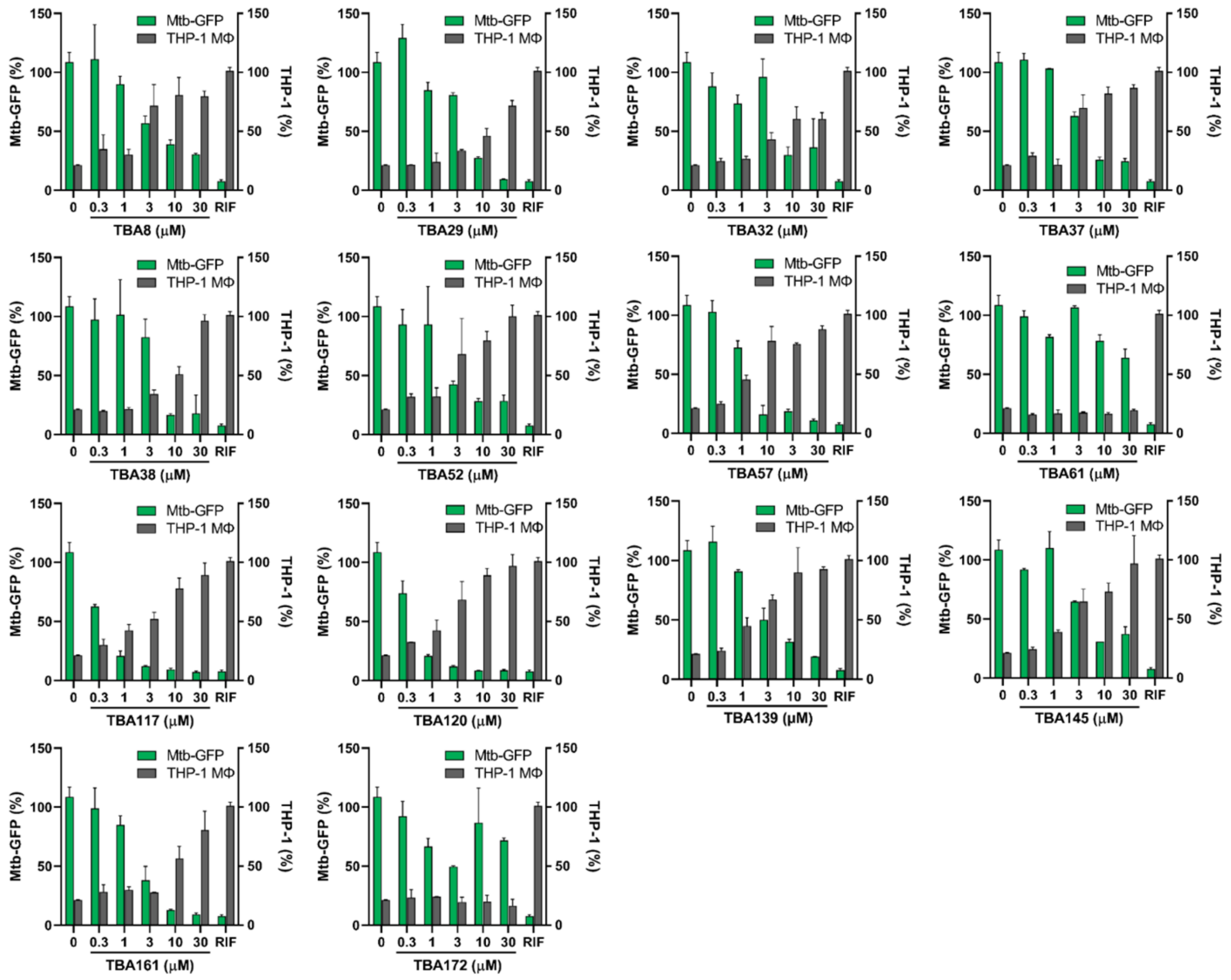

Fig. S4. Activity of TBA hit-compounds in the macrophage infection model. TBA hit compounds from zebrafish-infection model were tested for their intracellular activity in infected macrophages. THP-1 macrophages were infected with Mtb carrying pTetDuo, expressing *gfp* under control of a ATc -inducible promoter and *tdTomato* under the constitutive promoter. Infected macrophages were treated with various concentrations of each test compound for 5 days. The *gpf* expression was induced by the addition of ATc and macrophage nuclei were stained with Hoechst dye to detect macrophages (grey bars). The GFP signal within each macrophage was quantified, representing the amount of viable bacteria (green bars). DMSO and rifampicin (RIF, 3µM) treated samples served as a negative and positive control, respectively. Data points represent the average of duplicates with the standard deviations.

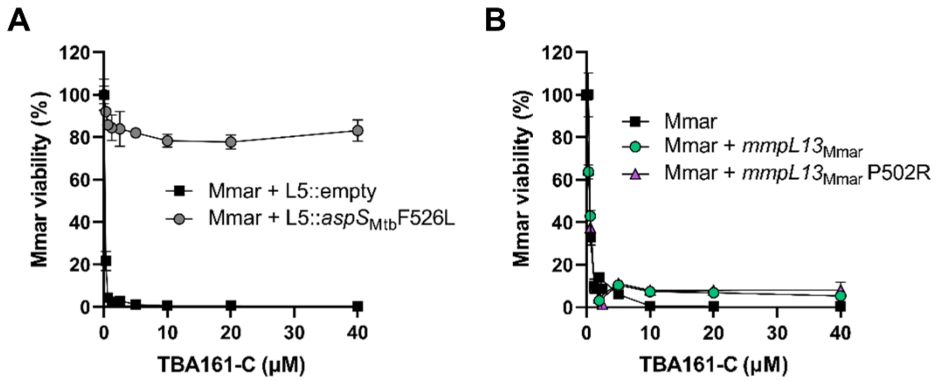

Fig. S5. Validation of mutations involved in TBA161-C resistance (A) *M. marinum* WT transformed with empty integrative plasmid pML1342 (Mmar + L5::empty) and *M. marinum* WT transformed with pML1342-*aspS*$_{Mtb}$F526L (Mmar + L5::*aspS*$_{Mtb}$F526L) were incubated with compound TBA161-C for 4 days at indicated concentrations. Data is presented as mean of triplicates ± SD. **(B)** Susceptibility of *M.marinum* WT (Mmar) and *M.marinum* WT transformed with pMN016-*mmpL13* $_{Mmar}$ (Mmar + *mmpL13*) and pMN016-*mmpL13*$_{Mmar}$ P502R (Mmar + *mmpL13* P502R) was measured after 4 days of incubation with TBA161-C. Data is presented as mean of duplicates ± SD.

Disease Models & Mechanisms • Supplementary information

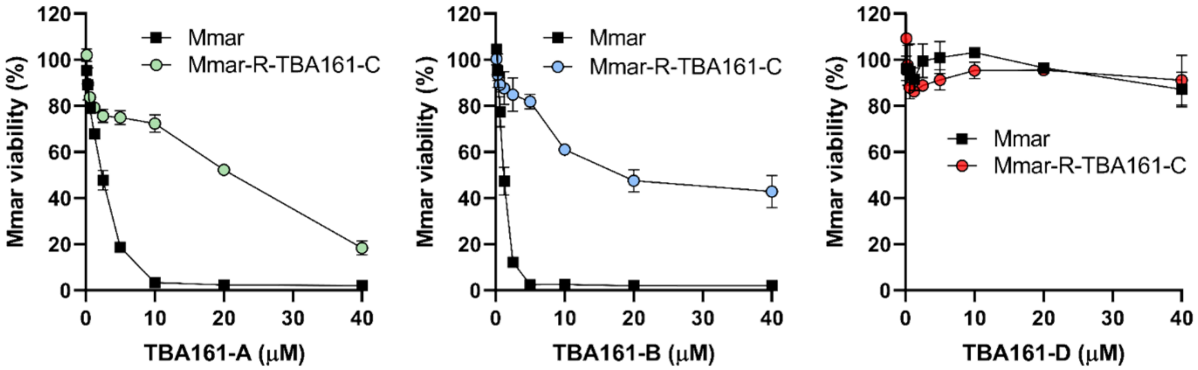

Fig. S6. TBA161-A and TBA161-B exhibit cross-resistance to TBA161-C resistant *M. marinum* strains. Susceptibility of *M. marinum* WT strain (Mmar) and TBA161-C resistant isolate (Mmar-R-TBA161-C) after 4 days of incubation with TBA161-A, TBA161-B, TBA161-D. Data is presented as mean of duplicates ± SD.

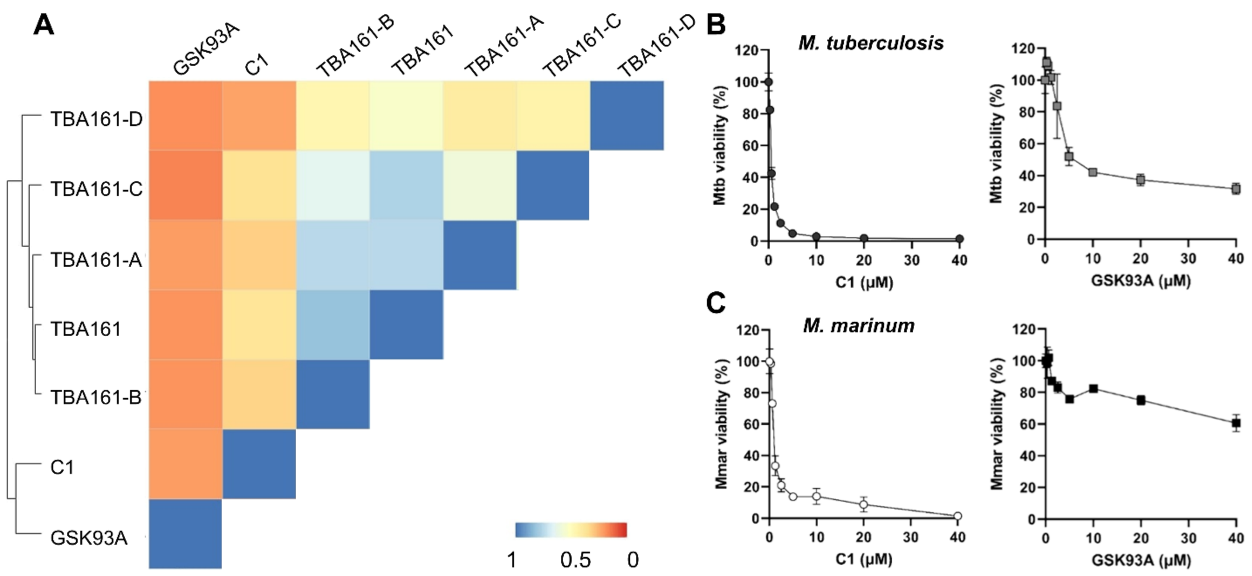

Fig. S7. Different AspS inhibitors exhibit low structure similarity and dissimilar activity. (A) Tanimoto coefficient graph comparing structure similarities of different TBA variants (TBA161, TBA161-A, TBA161-B, TBA161-C, TBA161-D) and AspS inhibitors (C1, GSK93A), created using ChemMine Tools (Backman et al., 2011). Value 0 describes the lowest similarity, and value 1 describes the highest similarity (identical). **(B)** Susceptibility of *M.marinum* strain towards compounds C1 or GSK93A after 4 days of incubation. Data is presented as mean of duplicates ± SD. **(C)** Susceptibility of Mtb WT strain towards compounds C1 or GSK93A after 7 days of incubation. Data is presented as mean of duplicates ± SD.

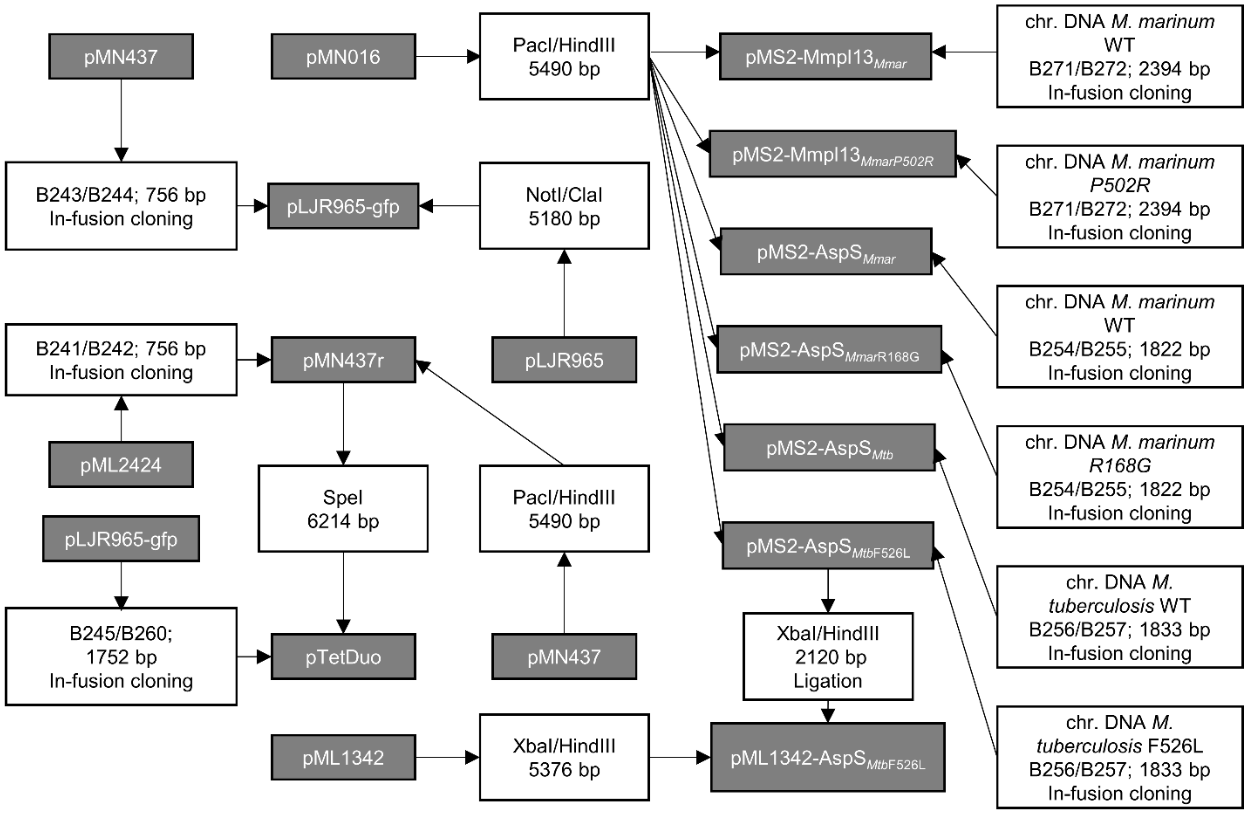

Fig. S8. Cloning strategy for new plasmids used in this study. Constructed plasmids are included in grey boxes. The primer pairs used for PCR amplification and correlated restriction enzymes for cloning are listed in white boxes. The DNA template for the PCR reactions is listed above the primer pairs. If several primer pairs are listed, overlap PCR was used to fuse the PCR fragments. When a DNA fragment was obtained by digestion of a plasmid, the utilized restriction enzymes and the length of the obtained fragments are indicated. Constructed primers with their sequences and plasmids with their features and are listed in Tables S5 and S6, respectively.

Table S1. Minimal inhibitory concentration (MIC) of antibiotics against *M. marinum*, *E. coli* and *S.pneumoniae*. MIC_{90} values represent the concentration required to inhibit 90% of bacterial growth. Not determined: n.d.

	**MIC_{90} (µM)**		
**Antibiotic**	***M. marinum***	***E. coli***	***S. pneumoniae***
Ceftriaxone	n.d.	0.19	0.05
Ceftazidime	n.d.	n.d.	0.91
Kanamycin	2	n.d.	n.d.
Levofloxacin	1.3	0.35	5.53
Linezolid	4	n.d.	n.d.
Meropenem	n.d.	0.14	0.29
Penicillin	n.d.	n.d.	0.04
Streptomycin	3	n.d.	n.d.

Table S2. Activity of TBA161-C against selected microorganism, cytotoxicity and zebrafish embryo toxicity. MIC_{90} values represent minimal inhibitory concentration required to inhibit 90% of bacterial growth. TD_{50} values represent median toxic dose, a dose required to kill half the members of a tested population.

**Organism / cell line**	**MIC_{90} (µM)**	**TD_{50} (µM)**
Escherichia coli K12	> 80	
Bacillus subtilis 168	> 80	
Klebsiella pneumoniae LMG20218	> 80	
Acinetobacter baumannii LMG01041	> 80	
Acinetobacter baumannii 1757	> 80	
Mycobacterium abscessus 144C	> 40	
Mycobacterium abscessus RIVM	> 40	
THP-1 cell line		> 40
RAW 264.7 cell line		> 40
Danio rerio embryos		> 40
Danio rerio embryos (1% DMSO)		> 80

Disease Models & Mechanisms • Supplementary information

Table S3A. Single nucleotide polymorphisms identified in TBA161-C resistant *M. marinum-tdTomato* mutants.

Strain	Position	Count/Coverage	Gene mutation	Amino acid change	Gene
R1/R2/R3	5296034	193/194	G-C	P-R (502)	MMAR_4305 (*mmpL13*)
R1/R2/R3	25992266	173/174	C-G	R-G (168)	MMAR_2158 (*aspS*)

Table S3B. Single nucleotide polymorphisms identified in TBA161-C resistant *M. tuberculosis* mutants.

Strain	Position	Count/Coverage	Gene mutation	Amino acid change	Gene
R1	636175	701/702	Deletion (C)	Frameshift	Rv0544c
R1	2896236	705/707	G-C	F-L(526)	Rv2572c (*aspS*)
R1	204312	692/711	Deletion (A)	Frameshift	Rv0173 (*lprK*)

Table S4. Strains used in this study.

Strain	Characteristics	References
Acinetobacter baumannii 1757	Clinical isolate	This study
Acinetobacter baumannii LMG01041	Laboratory strain	(Li et al., 2018)
Bacillus subtilis 168	Laboratory strain	ATCC 23857
Escherichia coli DH5α	*rec*A1; *end*A1; *gyr*A96; *thi*; *rel*A1; *hsd*R17(r_K^-,m_K^+); *sup*E44; ϕ80Δ*lacZ*ΔM15; Δ*lacZ*(YA-argF)UE169	(Sambrook et al., 1989)
Escherichia coli K12	Laboratory strain	ATCC 47076
Escherichia coli GSK1161434	Clinical isolate	GSK Microbiology Culture Collection(O'dwyer et al., 2015)
Klebsiella pneumoniae LMG20218	Laboratory strain	(Li et al., 2018)
Mycobacterium abscessus 144C	Clinical isolate	This study
Mycobacterium abscessus RIVM	Clinical isolate	This study
Mycobacterium marinum M^{USA}	Laboratory strain	ATCC BAA-535
Mycobacterium marinum R-TBA161-C	M^{USA} derivative, *aspS*$_{R168G}$ (*mmar_2158*)	This study
Mycobacterium tuberculosis H37Rv	Laboratory strain	ATCC 25618
Mycobacterium tuberculosis R-TBA161-C	H37Rv derivative, *aspS*$_{F526L}$ (*rv2572c*)	This study
Streptococcus pneumoniae D39V	Serotype 2	(Avery et al., 1944; Slager et al., 2018)

Disease Models & Mechanisms • Supplementary information

Table S5. Primers used in this study.

Oligonucleotide	Sequence 5'-3'
B255	ATCCGCATGCTTAATTAAGGGAGAACGTGTTTGTGCTGCGTAGCCA
B254	ATTAATTAGCTAAAGCTTATGTCCCCTCAACTTGTTTGG
B256	ATCCGCATGCTTAATTAAGGGAGAACGTGTTTGTGCTGCGCAGCCA
B257	ATTAATTAGCTAAAGCTTATGCCTGCTGGACCCGCTTG
B245	CTTAGCTAATCAACTAGTGTTAACTATTTAATTGGGGACCC
B260	AATGCAGCTAGAACTAGTTCTGACCAGGGAAAATAGCCCTC
B243	AGAGAAGGCGGTATCGATATGTCGAAGGGCGAGGAGCT
B244	CTAATCAGCGGCCGCACGCGTCTACTTGTACAGCTCGTCCATGCC
B242	ATTAATTAGCTAAAGCTTACTTGTACAGCTCGTCCATGC
B241	ATCCGCATGCTTAATTAACAGAAAGGAGGTTAATAATGGTGAGCAAGGGCGA
B271	GCACGATCCGCATGCTTAATTAAGGGAGAACATGTTGCAGGGGATCGCTCG
B272	CCAATTAATTAGCTAAAGCTCTATCCACGACCACTCAGCG

Disease Models & Mechanisms • Supplementary information

Table S6. Plasmids used in this study.

Plasmids	Characteristics	References
pMS2	p_{hsp60}; oriE(ColE1); PAL5000 origin, *hyg*^{R}; 5229 bp	(Kaps et al., 2001)
pMS2-tdTomato	p_{wmyc}-*tdTomato*; oriE(ColE1); PAL5000 origin; *hyg*^{R}; 6132 bp	(Ho et al., 2021)
pMN016	p_{smyc}-mspA; ColE1 origin; PAL5000 origin; *hyg;* 6164 bp	(Stephan et al., 2005)
pLJR965	L5 attP, *aph*^{R}*, tetR*^{on},p_{teto}-*Sth1 dCas9,* oriE, 8631 bp	(Rock et al., 2017)
pLJR965-gfp	L5 attP, *aph*^{R}*, tetR*^{on},p_{teto}-*mgfp2+,* oriE, 5909 bp	This study
pMN437	p_{smyc}-*mycgfp2+,* oriE(ColE1); PAL5000 origin, *hyg*^{R}; 6236 bp	(Song et al., 2008)
pMN437R	p_{smyc}-*tdtomato,* oriE(ColE1); PAL5000 origin, *hyg*^{R}; 6214 bp	This study
pTetDuo	PAL5000 origin, *hyg*^{R}*, tetR*^{on},p_{teto}-*mgfp2+,* p_{smyc}-*tdtomato,* oriE, 7936bp	This study
pML2424	pUC origin; pAL5000ts; *sacR*; *sacB*; p_{wmyc}-*tdtomato* ; *loxP*-p_{smyc}-*mycgfp2+- hyg -loxP*; 9527bp	(Ofer et al., 2012)
pMS2-AspS$_{Mmar}$	p_{smyc}-AspS$_{Mmar}$, oriE(ColE1); PAL5000 origin; *hyg*^{R}; 7282 bp	This study
pMS2-AspS$_{MmarR168G}$	p_{smyc}-AspS$_{MmarR168G}$, oriE(ColE1); PAL5000 origin; *hyg*^{R}; 7282 bp	This study
pMS2-AspS$_{Mtb}$	p_{smyc}-AspS$_{Mtb}$, oriE(ColE1); PAL5000 origin; *hyg*^{R}; 7291 bp	This study
pMS2-AspS$_{MtbF526L}$	p_{smyc}-AspS$_{MtbF526L}$, oriE(ColE1); PAL5000 origin; *hyg*^{R}; 7291 bp	This study
pML1342	p_{wmyc}-xylEM; oriE(ColE1); *hyg*^{R}; *int L5*; *ttsbiA*; *ttsbiB;* 5404 bp	(Huff et al., 2010)
pML1342-AspS$_{MtbF526L}$	p_{wmyc}-xylEM; p_{smyc}-AspS$_{MtbF526L}$; oriE(ColE1); *hyg*^{R}; *int L5*; *ttsbiA*; *ttsbiB;* 7496 bp	This study
pMS2-MmpLl13$_{Mmar}$	p_{smyc}-Mmpl13$_{Mmar}$, oriE(ColE1); PAL5000 origin; *hyg*^{R}; 7844 bp	This study
pMS2-MmpLl13$_{MmarP502R}$	p_{smyc}-Mmpl13$_{MmarP502R}$, oriE(ColE1); PAL5000 origin; *hyg*^{R}; 7844 bp	This study

Disease Models & Mechanisms • Supplementary information

Table S7. Physicochemical properties of non-toxic TBA compounds and reference antibiotics.

Click here to download Table S7

Supplementary references

Avery, O. T., Macleod, C. M. and McCarty, M. (1944). Studies on the chemical nature of the substance inducing transformation of pneumococcal types: Induction of transformation by a desoxyribonucleic acid fraction isolated from pneumococcus type iii. *J. Exp. Med.* **79**, 137–158.

Backman, T. W. H., Cao, Y. and Girke, T. (2011). ChemMine tools: An online service for analyzing and clustering small molecules. *Nucleic Acids Res.* **39**,.

Bertz, S. H. (2002). The first general index of molecular complexity. *J. Am. Chem. Soc.* **103**, 3599–3601.

Hendrickson, J. B., Huang, P. and Toczko, A. G. (1987). Molecular Complexity: A Simplified Formula Adapted to Individual Atoms. *J. Chem. Inf. Comput. Sci.* **27**, 63–67.

Ho, V. Q. T., Verboom, T., Rong, M. K., Habjan, E., Bitter, W. and Speer, A. (2021). Heterologous expression of ethA and katG in Mycobacterium marinum enables the rapid identification of new prodrugs active against Mycobacterium tuberculosis. *Antimicrob. Agents Chemother.* **65**,.

Huff, J., Czyz, A., Landick, R. and Niederweis, M. (2010). Taking phage integration to the next level as a genetic tool for mycobacteria. *Gene* **468**, 8–19.

Kaps, I., Ehrt, S., Seeber, S., Schnappinger, D., Martin, C., Riley, L. W. and Niederweis, M. (2001). Energy transfer between fluorescent proteins using a co-expression system in Mycobacterium smegmatis. *Gene* **278**, 115–124.

Li, Q., Montalban-Lopez, M. and Kuipers, O. P. (2018). Increasing the antimicrobial activity of nisinbased lantibiotics against Gram-negative pathogens. *Appl. Environ. Microbiol.* **84**,.

O'dwyer, K., Spivak, A. T., Ingraham, K., Min, S., Holmes, D. J., Jakielaszek, C., Rittenhouse, S., Kwan, A. L., Livi, G. P., Sathe, G., et al. (2015). Bacterial Resistance to Leucyl-tRNA Synthetase Inhibitor GSK2251052 Develops during Treatment of Complicated Urinary Tract Infections. *Antimicrob Agents Chemother*.

Ofer, N., Wishkautzan, M., Meijler, M., Wang, Y., Speer, A., Niederweis, M. and Gur, E. (2012). Ectoine biosynthesis in Mycobacterium smegmatis. *Appl. Environ. Microbiol.* **78**, 7483–7486.

Disease Models & Mechanisms • Supplementary information

Rock, J. M., Hopkins, F. F., Chavez, A., Diallo, M., Chase, M. R., Gerrick, E. R., Pritchard, J. R., Church, G. M., Rubin, E. J., Sassetti, C. M., et al. (2017). Programmable transcriptional repression in mycobacteria using an orthogonal CRISPR interference platform. *Nat. Microbiol.* **2**, 16274.

Sambrook, J., Fritsch, E. F. and Maniatis, T. (1989). Molecular cloning: a laboratory manual. *Mol. cloning a Lab. manual.*

Slager, J., Aprianto, R. and Veening, J. W. (2018). Deep genome annotation of the opportunistic human pathogen Streptococcus pneumoniae D39. *Nucleic Acids Res.* **46**, 9971–9989.

Song, H., Sandie, R., Wang, Y., Andrade-Navarro, M. A. and Niederweis, M. (2008). Identification of outer membrane proteins of Mycobacterium tuberculosis. *Tuberculosis* **88**, 526–544.

Stephan, J., Bender, J., Wolschendorf, F., Hoffmann, C., Roth, E., Mailänder, C., Engelhardt, H. and Niederweis, M. (2005). The growth rate of Mycobacterium smegmatis depends on sufficient porin-mediated influx of nutrients. *Mol. Microbiol.* **58**, 714–730.
